# Genome-Wide Association Study for Resistance to Tan Spot in Synthetic Hexaploid Wheat

**DOI:** 10.3390/plants11030433

**Published:** 2022-02-05

**Authors:** Nerida Lozano-Ramírez, Susanne Dreisigacker, Carolina P. Sansaloni, Xinyao He, Sergio Sandoval Islas, Paulino Pérez-Rodríguez, Aquiles Carballo Carballo, Cristian Nava-Díaz, Masahiro Kishii, Pawan K. Singh

**Affiliations:** 1 International Maize and Wheat Improvement Center (CIMMYT), Km. 45 Carretera México-Veracruz, El Batán, Texcoco 56237, Mexico; n.lozano@cgiar.org (N.L.-R.); c.sansaloni@cgiar.org (C.P.S.); x.he@cgiar.org (X.H.); m.kishii@cgiar.org (M.K.); 2 Colegio de Postgraduados (COLPOS), Montecillo 56264, Mexico; sersando.islas@gmail.com (S.S.I.); perpdgo@gmail.com (P.P.-R.); aquiles.carballo@gmail.com (A.C.C.); cnava@colpos.mx (C.N.-D.)

**Keywords:** *Aegilops tauschii*, durum wheat, synthetic hexaploid wheat, tan spot, genome-wide association study

## Abstract

Synthetic hexaploid wheat (SHW) has shown effective resistance to a diversity of diseases and insects, including tan spot, which is caused by *Pyrenophora tritici-repentis*, being an important foliar disease that can attack all types of wheat and several grasses. In this study, 443 SHW plants were evaluated for their resistance to tan spot under controlled environmental conditions. Additionally, a genome-wide association study was conducted by genotyping all entries with the DArTSeq technology to identify marker-trait associations for tan spot resistance. Of the 443 SHW plants, 233 showed resistant and 183 moderately resistant reactions, and only 27 were moderately susceptible or susceptible to tan spot. Durum wheat (DW) parents of the SHW showed moderately susceptible to susceptible reactions. A total of 30 significant marker-trait associations were found on chromosomes 1B (4 markers), 1D (1 marker), 2A (1 marker), 2D (2 markers), 3A (4 markers), 3D (3 markers), 4B (1 marker), 5A (4 markers), 6A (6 markers), 6B (1 marker) and 7D (3 markers). Increased resistance in the SHW in comparison to the DW parents, along with the significant association of resistance with the A and B genome, supported the concept of activating epistasis interaction across the three wheat genomes. Candidate genes coding for F-box and cytochrome P450 proteins that play significant roles in biotic stress resistance were identified for the significant markers. The identified resistant SHW lines can be deployed in wheat breeding for tan spot resistance.

## 1. Introduction

Diseases are major threats that significantly reduce yield when crops are grown under disease-favoring conditions. Wheat foliar diseases have gained increased importance in recent years due to various factors such as the adoption of conservation agriculture practices, commercial cultivation of susceptible varieties, and high-evolution dynamics of the causal pathogens [1]. Furthermore, climate change often results in severe disease epidemics that significantly limit grain yield and quality in wheat [2]. About 12–14% of the global wheat production is lost each year due to diseases [3]. The causative agents of these diseases, mainly fungal pathogens, infect multiple wheat tissues such as root, stem, leaf, spike, and grain. Based on the frequency and severity levels of disease epidemics, the diseases that infect leaf and spike/grain are considered of greater importance. In this sense, many researchers agree that “stripe rust” caused by *Puccinia striiformis* f. sp. *tritici*; “tan spot” by *Pyrenophora tritici-repentis* (Died.) Drechs. (anamorph *Drechslera tritici-repentis* (Deceased) Shoem.); “Septoria nodorum blotch” by *Parastagonospora nodorum* (syn. ana. *Stagonospora*; teleo. *Phaeosphaeria*) (Berk.) Quaedvlieg, Verkley & Crous, and “Septoria tritici blotch” by *Mycosphaerella graminicola* (Fuckel) Schroeter, in Cohn (anamorph *Zymoseptoria tritici* Rob ex Desm.) are some of the most important foliar diseases [3,4].

Tan spot (synonymous with yellow spot) pathogen *P. tritici-repentis* belongs to the order of dothideomycete in ascomycete [5] and can attack durum and bread wheat, as well as many other grass species. This foliar wheat disease is found globally, with symptoms mainly including necrosis and chlorosis on leaf tissues, reducing the photosynthetic area, and resulting in poor grain filling, kernel shriveling, a reduced number of kernels per head, and severe yield losses [6]. Yield losses of up to 49% have been attributed to tan spot under favorable disease conditions [6]. Additionally, the disease can lead to reductions in grain quality by forming red or pink smudge. The pathogen-induced lesions may coalesce and cover most, or the entirety of, the leaf surface; these symptoms are associated with the fungal-produced necrotrophic effectors (NEs), previously known as host-selective toxins (HSTs) [7]. The necrosis and chlorosis associated with tan spot result from toxins produced by the pathogen as initially proven by Tomas and Bockus [8] and Lamari and Bernier [9]. Currently, eight races of *P. tritici-repentis* have been identified based on symptoms of necrosis and chlorosis on a set of differential wheat varieties/lines [10].

Due to the overwintering habit of *P. tritici-repentis* on crop residues or stubbles, tan spot is a major concern in sustainable zero-tillage agricultural systems. The disease cycle consists of a primary infection caused by fungal ascospores at the beginning of the growing season, and numerous subsequent infections by fungal conidia throughout the growing season. Although the disease can be controlled using cultural and/or chemical methods, host resistance against tan spot is the most cost-effective and environmentally friendly way to limit yield losses.

To identify novel and more effective sources of resistance, breeding programs have explored synthetic hexaploid wheat (SHW) that harbors a broad spectrum of resistance to diseases and insects [11]. SHW (2n = 6x = 42, AABBDD) derives from a cross between modern durum wheat (2n = 4x = 28, AABB, *T. turgidum* L.) and wild goat grass (2n = 2x = 14, DD, *Ae. tauschii* Coss.). SHW is considered as an ideal bridging germplasm for the transfer of desirable genes from DW and *Ae. tauschii* to bread wheat [12].

The genome-wide association study (GWAS) explores linkage disequilibrium (LD) in a collection of varieties or accessions [13,14,15,16] and is a powerful tool to identify quantitative trait loci (QTL). It uses recombination events that occurred during the history of variety development, resulting in an often-improved genetic resolution for identifying QTL compared to bi-parental mapping populations, which have usually undergone only one or a few generations of recombination. In addition, GWAS allows for the screening of a large number of lines for a whole spectrum of traits. GWAS has been applied to identify genomic regions associated with tan spot resistance in common wheat. Gurung et al. [17] conducted the first GWAS for tan spot resistance in a spring common wheat landrace collection and found QTL on chromosomes 1D, 2A, 2B, 2D, 4A, 5B and 7D for race 1 and on chromosomes 1D, 2B, 2D and 7D for race 5. Furthermore, GWAS has been performed with different races of *P. tritici-repentis* in panels of spring wheat landraces [18] and with unknown races on a European winter wheat collection [19]. Multiple races were used for a GWAS in a collection of North American winter wheat cultivars and breeding lines [20], and race 1 isolates in the Vavilov wheat collection at both seedling and adult stages [21]. The QTL identified in those studies corresponded partly to the NE sensitivity loci and previously reported loci, whereas others were novel.

A few GWAS studies have also been performed to identify significant markers related to tan spot resistance in CIMMYT (International Maize and Wheat Improvement Center) wheat germplasm [22,23,24]. Singh et al. [22] indicated the association of tan spot resistance with markers on multiple A- and B-genome chromosomes. Similarly, Juliana et al. [23] identified 14 markers on A- and B-genome chromosomes. Phuke et al. [24] performed GWAS on a panel of South Asian and CIMMYT spring bread wheat genotypes and found significant markers on chromosomes 1B, 2A, 2B, 3B, 4A, 5A, 5B, 6A, and 7D. However, none of these studies included SHW.

The current GWAS study was conducted on a diverse panel of 443 SHW plants in order to (1) evaluate their resistance to tan spot under controlled environmental conditions and (2) identify possible new genomic regions for tan spot resistance.

## 2. Results

### 2.1. Resistance to Tan Spot at the Seedling Stage

Uniform and consistent tan spot development was observed during seedling evaluation in the greenhouse. Analyses of variance (ANOVA) showed significant differences among SHW plants (*p* < 0.001) for reaction to tan spot. The checks Erik, Glenlea, 6B-662, and 6B-365 displayed scores of 1.0, 4.8, 2.5 and 3.4, respectively (Table 1), verifying the identity of *P. tritici-repentis* and successful inoculation.

**Figure 1:**
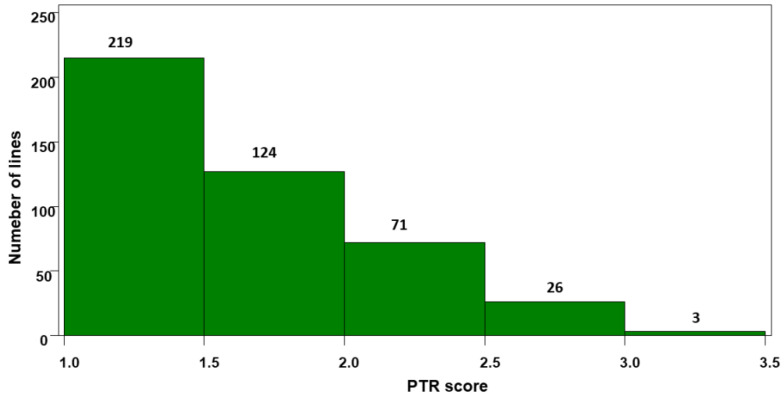
Histograms of tan spot disease scores for the 443 synthetic hexaploid wheat accessions.

Most SHW plants displayed resistant and moderately resistant reactions (Supplementary Table S1). Out of the 443 SHW plants, 219 (49.4%) showed resistance (R) and 195 (44.0%) moderate resistance (MR) with disease scores of 1.5 to 2.5 that were comparable to the resistant check Erik and the moderately resistant check 6B-662. Only 29 SHW plants (6.5%) were moderately susceptible (MS) with disease scores of 2.6 to 3.5 that were still better than the susceptible check Glenlea and 6B-365 (Table 1, and Figure 1).

Of the 40 DW parents, six (15%) had reaction scores of 1.0–1.5 (R) and 12 (30%) had reactions scores of 1.6–2.5 (MR), developing mostly small dark to maroon lesions on the leaves. Twenty-two entries (55%) were observed to have a mean reaction score between 2.6 and 4.3, being considered MS to S, wherein large necrotic lesions with or without chlorosis was observed. (Table 1, Supplementary Table S1).

### 2.2. Genome-Wide Association Mapping under Different References Maps

Using the markers mapped on the 100K consensus map, the first two principal components (PCs) separated two clear groups of entries of similar sizes and some entries in between, explaining around 34% of the total variability (Supplementary Figure S1). As described in the Section 4, possible population structure was controlled by fitting the first five PCs from the correlation matrix as a fixed variate. In addition, the coefficient of parentage used as a random variable for fitting the GWAS mixed linear model (MLM) effectively controlled the remnant population structure after fitting the first three PCs.

**Figure 2:**
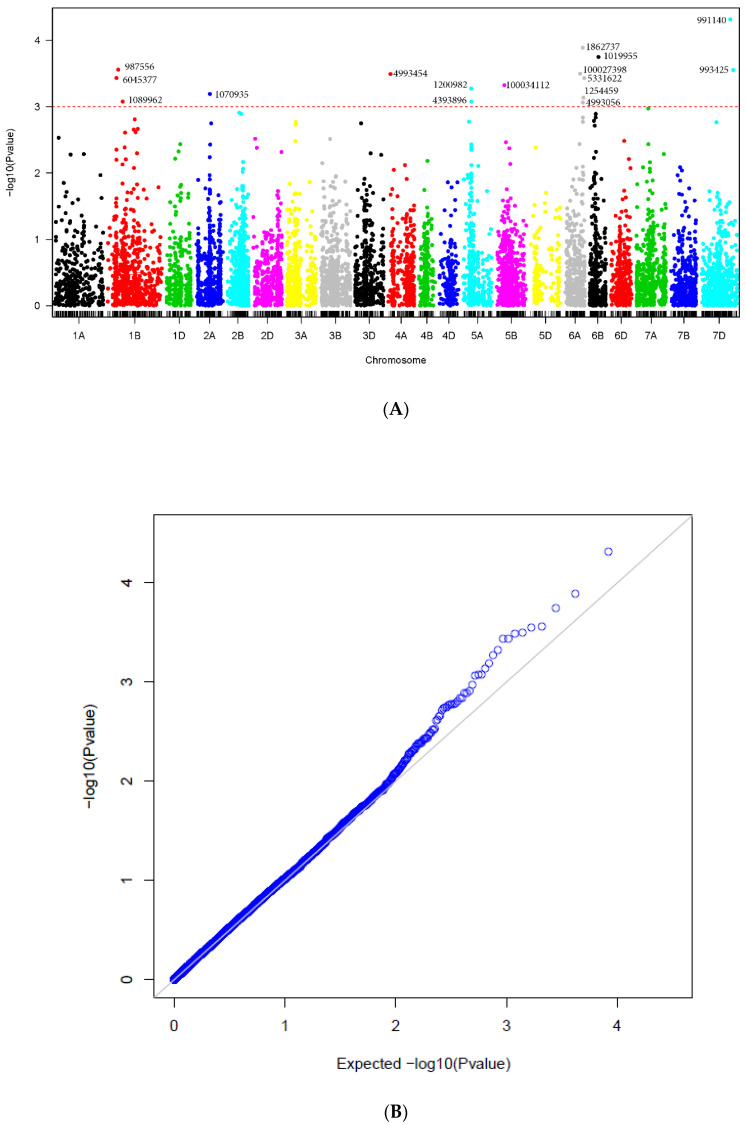
(**A**) Manhattan plots for tan spot disease corresponding to the Consensus Map. The *p*-values are shown on a log10 scale. The marker is considered significant if log10 scale is 3 or higher. (**B**) QQplot displaying the quantiles of the empirical distribution (blue circles) of the results obtained in this study with those of the distribution that we would expect theoretically if the null hypothesis is true (black line).

Significant marker-trait associations detected using the consensus map are shown in Table 2 and Figure 2. The 16 significant markers were located on chromosomes 1B (3), 2A (1), 4A (1), 5A (2), 5B (1), 6A (5), 6B (1) and 7D (2). The markers with the highest allele substitution effects were located on chromosomes 4A (−0.55), 6B (−0.44), and 7D (0.59).

**Figure 3:**
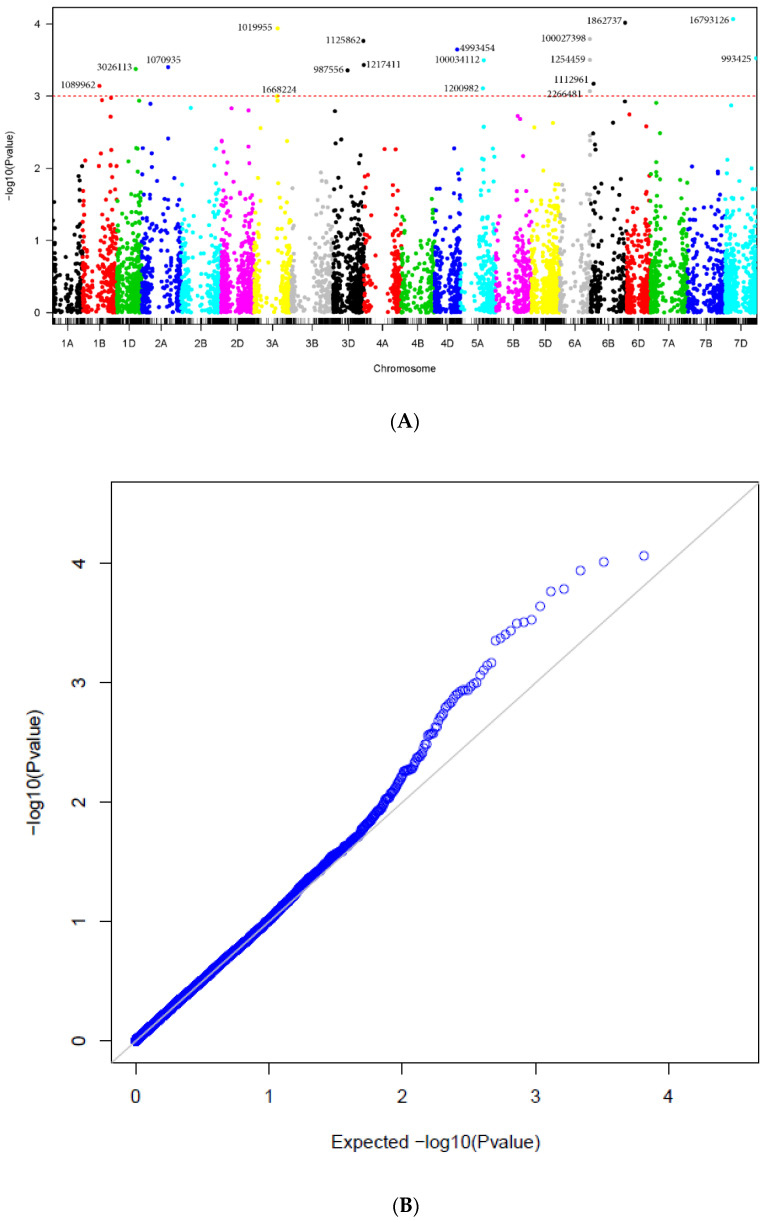
(**A**) Manhattan plots for tan spot disease corresponding to the Physical position (Chinese spring Ref Seq ver.1.0). The *p*-values are shown on a log10 scale. The marker is considered significant if log10 scale is 3 or higher. (**B**) QQplot displaying the quantiles of the empirical distribution (blue circles) of the results obtained in this study with those of the distribution that we would expect theoretically if the null hypothesis is true (black line).

Significant marker-trait associations when markers were aligned to the whole genome sequence of Chinese Spring (CS, IWGSC RefSeq v1.0) are shown in Table 3 and Figure 3. The 18 significant markers were located on chromosomes 1B (1), 1D (1), 2A (1), 3A (2), 3D (3), 4D (1), 5A (2), 6A (3), 6B (2) and 7D (2). Ten of the markers overlapped with those presented in Table 2, out of which six exhibited the same chromosome assignments on the genetic and physical maps, whereas four showed different chromosome assignments (yet mainly homologous chromosomes) on the two maps. The markers with the highest allele substitution effects were located on chromosomes 3A (−0.44), 4D (−0.56), and 7D (0.61).

**Figure 4:**
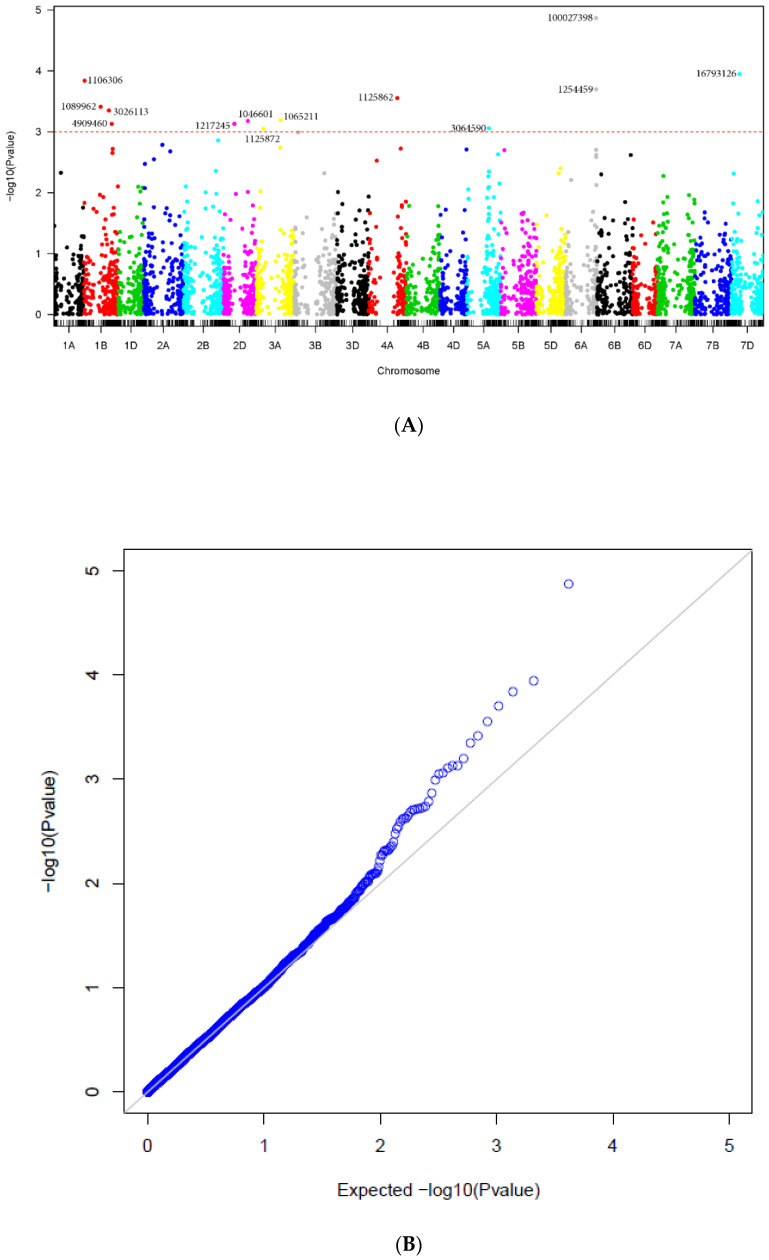
(**A**) Manhattan plots for tan spot disease corresponding to the Durum Wheat (cv. Svevo) and *Ae. tauschii* reference genomes (Ref Seq Rel. 1.0). The *p*-values are shown on a log10 scale. The marker is considered significant if log10 scale is 3 or higher. (**B**) QQplot displaying the quantiles of the empirical distribution (blue circles) of the results obtained in this study with those of the distribution that we would expect theoretically if the null hypothesis is true (black line).

Thirteen markers were significantly related to tan spot resistance, aligned to the durum wheat cultivar Svevo and the *Ae. tauschii* reference genomes. These markers were located on chromosomes 1B (4), 2D (2), 3A (2), 4A (1), 5A (1), 6A (2) and 7D (1) (Table 4 and Figure 4). Only three markers from Table 4 coincided with the significant markers found in Table 2 and Table 3. Marker 3026113 on chromosome 1B in Svevo was found to be significant on chromosome 1D aligned to the physical map of CS. Similarly, marker 1125862 on chromosome 3A in Svevo aligned to chromosome 3D in the physical map of CS (Table 3). Marker 16793126 aligned to chromosome 7D in the *Ae. tauschii* and CS physical maps (Table 3)**.** The markers with the highest allele substitution effects ranged from −0.20 to −0.27 and were located on chromosomes 1B, 3A, 5A, and 6A.

### 2.3. Comparison of the Significant Markers across the Different Maps

Table 5 summarizes the 30 genomic regions identified with different maps. A re-alignment of the sequences to the ABD, AB and D genomes could verify the physical position of several of the significant SNPs. Furthermore, 16 SNPs were found within annotated high-confidence gene sequences. Eight of these 16 possible candidate genes were annotated in the CS reference genome, four in Svevo and the residual four in the *Ae. tauschii* reference genome (Supplementary Table S2).

### 2.4. Marker-Trait Associations and QTL for Tan Spot Resistance

The allele frequency correlations (R^2^) among the markers were used to estimate LD. Based on the physical positions of observed marker-trait association in the CS reference genome, three potential QTL were identified on each of the chromosomes 3A, 5A and 6A. Out of the four significant markers on chromosome 3A, with marker IDs 1125872, 1668224, 1019955, and 1065211, the latter two markers were positioned at 474,447,292 Mb and 474,447,226 Mb, respectively, only 66 bp apart with a R^2^ of 0.89 and a significant LD *p*-value of 8.62E-16. The third marker (ID 1668224), despite being located 5.9 Mb apart from the previous two, still had R^2^ values of 0.87 and 0.89 and significant LD *p*-values of 6.54E-16 and 2.30E-16, with the two SNPs, respectively. Therefore, these three markers can be considered for a single QTL for resistance to tan spot. Marker 112872, however, was located far from the markers mentioned above and must represent an independent QTL.

Likewise, two markers on chromosome 5A (100034112 and 3064590) and four markers on chromosome 6A (1254459, 2266481, 100027398, and 1862737) were located in LD and thus represented one same QTL, whereas all the remaining SNPs identified in our study represented independent QTL, due to their mutually unlinked physical positions.

## 3. Discussion

The development of genetically resistant wheat cultivars is an effective and environmentally friendly mechanism for the control of diseases such as tan spot. In the following subsections, we discuss the findings of this GWAS in relations to previous studies performed.

### 3.1. Tan Spot Resistance in SHW

Modern bread wheat cultivars have only a few broad-spectrum sources of resistance to the major foliar spotting diseases, such as tan spot [25], and great efforts have been made in recent decades to identify and introduce new sources of resistance. Despite the number of studies performed and published for wheat diseases, only a few included SHW. For example, Bhatta et al. [26] studied 125 SHW plants for their resistance to diseases and pests such as rust, crown rot, cereal cyst nematodes, and Hessian fly. To the best of our knowledge, so far, no GWAS was performed to evaluate SHW for tan spot resistance.

Our study indicates that SHW plants present considerable resistance to tan spot due to the diverse genetic backgrounds of these lines. The DW parents were mostly of reaction types of MS and S, suggesting that the resistance in the SHW was either derived from *Ae. tauschii* or through possible favorable epistatic interaction (activation) between A/B- and D-genomes.

### 3.2. Comparisons with Previous Studies

#### 3.2.1. Significant Markers Found in the D- Genome Chromosomes

Our study found significant marker-trait associations for tan spot resistance on chromosomes 1D (marker ID 3026113), 2D (marker IDs 1217275, 1046621), 3D (marker IDs 987556, 1125862, 1217411), 4D (marker ID 4993454) and 7D (marker IDs 16793126, 991140, 993425). Thus, this is the first study to detect several significant genomic regions to tan spot resistance in the D-genome, in addition to the few loci reported previously. Phuke et al. [24] found a significant marker on chromosome 7D located at 550,216,751 Mb in CS. The closest significant marker on chromosome 7D in this study (marker ID 993425) was positioned at 620,252,508 Mb, physically distant and suggesting that at least two of the three marker-trait associations on chromosome 7D in this study are novel. The physical position of the third marker 991140 in CS could not be determined.

Tadesse et al. [27] studied resistance to tan spot in segregating F_2:3_ derived populations of SHW using simple sequence repeat (SSR) markers. The authors found that loci *tsn*3*a*, *tsn*3*b* and *tsn*3*c* are all located in the vicinity of the marker *Xgwm*2a located on chromosome 3D. The physical distance of this SSR marker to the SNP markers in our study was difficult to determine. Gurung et al. [17] performed GWAS in spring wheat landraces using DArT markers to identify chromosome regions associated to tan spot race 1 and 5 resistances. The authors found significant markers, among others, on chromosomes 1D and 7D associated to tan spot race 1 and in regions of chromosomes 2D and 7D for tan spot race 5. Similar to the study by Tadesse et al. [27], genomic regions could not be compared as different genotyping platforms were used.

#### 3.2.2. Significant Markers Found at the A and B Genome Chromosomes

The present study found significant marker-trait associations on the A-genome chromosomes 2A (marker ID 10770935), 3A (marker IDs, 1125872, 1668224, 1019955, 1065211) and those forming a QTL on chromosome 6A (marker IDs, 1862737, 100027398, 1254459, 2266481, 4993056, 5331622). None of the marker-trait associations coincided with those reported by Juliana et al. [23], except on chromosome 3A. Marker 1125872 was located at 135,590,641 Mb in our study and the marker in Juliana et al. [23] at 182,028,651 Mb. In the B-genome chromosomes, we found significant marker-trait associations on chromosomes 1B (markers IDs, 1106306, 6045377, 1089962, and 4909460), 4B (marker ID, 4993454), 5A (marker IDs, 4393896, 1200982, 100034112, and 3064590), and 6B (marker ID, 1112961); none of them were reported by Juliana et al. [23].

Phuke et al. [24] also found several marker-trait associations in the A- and B-genomes. The authors found a significant marker on chromosome 2A but in a different position than the one found in this study. A significant locus on chromosome 1B mapped to a physical position at 465,584,555 Mb and was also distant from markers on chromosome 1B of this study located at 340,462,174 Mb and 558,561,647 Mb. Significant marker on chromosome 6A was located at 596,903,177 Mb and coincided with the QTL found in this study at 599,622,814 Mb, 601,233,092 Mb, 602,989,232 Mb, and 602,745,555 Mb, thus representing the same QTL. The marker located on chromosome 5A in Phuke et al. [24] mapped to the physical position of 597,291,565 Mb, whereas the markers identified in this study forming a QTL are located a distance apart, at 454,770,615 Mb, 471,723,681 Mb, and 470,186,523 Mb, thus likely presenting a novel QTL.

The study by Kokhmetova et al. [28] detected three significant loci on chromosome 1B within a range of 86.7-92.2 cM, not distant from marker ID 1089962 located at 83.6 cM in this study using the same 100K consensus map. Furthermore, the QTL on chromosome 6A were in proximity to the markers found by Kokhmetova et al. [28] on the same chromosome. Kalia et al. [29] performed bi-parental QTL mapping for resistance to tan spot race 1 in a population with a SHW parent and identified QTL only on the A-genome chromosomes 1A, 6A, and 7A. Because DArT markers were used in this study, the physical positions of the QTL were, once again, difficult to compare. Similarly, Chu et al. [30] identified QTL on chromosomes in the A- and B-genome (2A, 5A and 5B) in a bi-parental mapping population having a SHW parent. The authors hypothesized that the expression of tan spot resistance genes in DW is suppressed (or diluted) but are activated when DW is crossed with *Ae. tauschii*, which could be due to inter-locus interaction (epistasis effects) between loci on A/B- and D-genomes. In the current study, increased resistance in SHW in comparison to their direct DW parents supports this hypothesis.

### 3.3. Underlying Candidate Genes Based on Protein Annotation

Two markers, one on chromosome 5A (marker ID 3064590) positioned at 470,186,523 Mb and the other one located on chromosome 6A (marker ID 1862737) at 599,622,814 Mb, were of particular interest in this study as they were positioned within genes that code for disease resistance related proteins, i.e., TraesCS5A02G254500/TRITD5Av1G155700 (F-box protein) and TraesCS6A02G378800/TRITD6Av1G217060 (cytochrome P450).

Candidate genes TraesCS5A02G254500/TRITD5Av1G155700 code for F-box proteins that play a role in protein regulation and degradation, plant photoperiodic and hormone signaling transduction. A total of 1796 F-box proteins have been identified and classified in wheat [31], many of which have been related to biotic stresses, particularly to fungal pathogens. In addition, F-box proteins have been observed to affect the plant metabolism and the regulation of plant enzymes involved in several diverse cellular processes [31]. It has been found that the F-box proteins can act in different development stages in a wheat cultivar. The identification of candidate genes being related to specific disease resistance should offer an opportunity to further elucidate the biological functions of F-box genes and proteins in wheat.

The cytochrome P450 (CYP) enzyme in plants is involved in the biosynthetic pathway of phytoalexins that are synthetized by plants to deter hostile organisms [32]. This CYP enzyme plays an important role in the metabolism of herbicides as a key factor in providing tolerance to some species and thus selectively between crops and weeds. Plants encounter various biotic and abiotic factors at different stages of their growth and development, and the group of CYP enzymes is important in the synthesis of certain metabolites which play a fundamental part in the response to biotic stresses. The CYT enzymatic protein participates in the formation of numerous secondary synthetized metabolites that protect plants from biotic and abiotic stresses [33]. The mycotoxin deoxynivalenol (DON) is a virulent factor for the development of Fusarium head blight in wheat. A wheat cytochrome P450 subfamily was found on chromosomes 3A, 3B and 3D of the wheat genome that was activated in the wheat spikelets as a response to the mycotoxin DON [34].

## 4. Materials and Methods

### 4.1. Plant Material

A total of 443 SHW plants generated by the CIMMYT Wheat Wide Crosses Program throughout several years were evaluated (Supplementary Table S1). These SHW plants were selected from a group of 1,524 SHW plants for resistance to diseases such as Fusarium head blight, Septoria tritici blotch, and rusts and phenological traits such as plant height and days to heading. The SWH plants were derived from crosses involving 40 DW parents and 277 *Ae. tauschii* accessions, where the DW parents were used in 1 to 54 crosses and the *Ae. tauschii* accessions were used in 1 to 7 crosses (Supplementary Table S1).

### 4.2. Phenotypic Evaluation for Tan Spot

The disease screening for tan spot was carried out in a greenhouse in CIMMYT, El Batán, Mexico (19°31′ N, 98°50′ W, elevation 2249 m above sea level) in 2018–2019. In addition to the 443 SHW plants, the 40 DW parents were also evaluated, while the *Ae. tauschii* parents could not be screened due to their challenging phenology as a wild species. The SHW seeds were vernalized (7 days at 4°C) to break dormancy and to obtain an even germination. Greenhouse experiments were arranged in a randomized complete block design with 12 replicates for each of the SHW entries and eight replicates for the DW parents. Each entry in each experiment had four plants. Four entries with different levels of resistances were considered “checks”—Erik (resistant), Glenlea (susceptible), 6B-365 (moderately susceptible), and 6B-662 (moderately resistant)—grown in plastic trays as experimental units to derive mean values for subsequent analysis. The seedlings were grown under controlled conditions in a temperature of 22–25/16–18 °C (day/night) and with a 16 h photoperiod.

For the induction of disease, the Mexican *P. tritici-repentis* isolate CIMFU 531-Ptr1 (race 1), well characterized by the CIMMYT Wheat Pathology Laboratory, was used. This isolate produces ToxA, based on inoculation experiments with differential genotypes, infiltration experiments, and PCR with the ToxA specific marker (data not shown). The isolate was grown on V8-PDA media [9], and the conidia concentration for inoculation was adjusted to 4 × 10^3^ spores mL^−1^ using a Fuchs–Rosenthal counting chamber, with one drop of Tween 20 (a surfactant reagent) per 100 mL added to the spore suspension.

In the two-leaf stage, when the second leaf was fully expanded at two weeks after sowing, the seedlings were inoculated with a conidial suspension of the CIMFU 531-Ptr1 isolate until runoff. Subsequently, the trays were moved to a mist chamber (RH 100%, 21–22°C) to facilitate infection. After 24 h, the plants were transferred back to the greenhouse bench. Seedling response was evaluated seven days post inoculation following the 1–5 lesion rating scale developed by Lamari and Bernier [9]. The readings from 12 and 8 inoculation experiments of the SHW plants and DW parents, respectively, were used to calculate the average seedling response, which was used for subsequent statistical analysis. The scale used for the tan spot reaction was based on continuous data given by the mean of the replicates: 1.0–1.5 = Resistant (R); 1.6–2.5 = Moderately Resistant (MR); 2.6–3.5 = Moderately Susceptible (MS); 3.6–5.0=Susceptible (S).

### 4.3. Plant Genotyping

The genomic DNA was extracted from 10-day-old seedlings of each SHW line using the modified cetyltrimethylammonium bromide (CTAB) method described in the CIMMYT laboratory protocols [35]. The DArTseq^TM^ technology [36] was applied to all samples at the Genetic Analysis Service for Agriculture (SAGA) in CIMMYT, Mexico. DArTseq uses a complexity reduction method including two enzymes (PstI and HpaII) to create a genome representation of the samples. A PstI-RE site-specific adapter is then tagged with 96 different barcodes enabling the multiplexing of a 96-well microtiter plate with equimolar amounts of amplification products to run in an Illumina sequencer Novaseq6000 (Illumina Inc., San Diego, CA, USA). The successfully amplified fragments were sequenced up to 83 bases.

A pipeline developed by DArT P/L was used to generate allele calls for SNP and SilicoDArT (presence/absence variation markers) [36]. A 100K consensus map [37] was used to obtain genetic positions of the SNPs. To obtain the physical positions, sequence reads were aligned to the reference genome of Chinese Spring (CS) IWGSC Ref Seq v1.0 [38], the reference genome of DW cv. Svevo Ref Seq Rel. 1.0 [39] and the reference genome of *Ae. tauschii* (v.4, 2017) [40].

A total of 67,436 DArTSeq SNP markers were originally scored, out of which 50% (34,790) were aligned to the reference genomes. Filtering was carried out excluding SNP with <0.05 allele frequency and >20% missing data points. Finally, 5800 DArTSeq markers were retained and used for GWAS analysis. The allele substitution effects for the significant marker-trait association were estimated by the mean phenotypic differences of alleles making the assumption that one genotype has effects equal to zero. Marker sequences were re-aligned (BLASTn) to the diverse reference sequences using the Ensembl plant public website (https://plants.ensembl.org/, accessed on 2 February 2022) to verify the position of the SNPs.

### 4.4. Statistical Analysis and Genome-Wide Association Analysis

For the disease data, statistical analyses were performed using the Statistical Analysis System version 9.1 [41]. Analyses of variance (ANOVA) were conducted on the average reactions of the SHW, the DW parents and checks for tan spot. The Best Linear Unbiased Estimates (BLUE) were computed for each of the 443 SHW genotypes.

The BLUE for disease severity were used as an input to conduct GWAS using the TASSEL (Trait Analysis by Association Evolution and Linkage) software ver. 5 [42]. We used the mixed linear model (MLM) [43] to simultaneously include the level of relatedness based on marker data and identical by descent (IBD) computed from the coefficient of parentage, which controls population structure. Additionally, population structure was controlled by fitting the first three principal components (PC) from the kinship matrix taken as the fixed variate and the coefficient of parentage (COP) as the random variable. The false-discovery rate (FDR) was used to assess the significance of the *p*-value (<0.05). The allelic effects of the significant marker-trait associations were estimated as the difference between the mean value of lines, with and without the favorable alleles, and were presented as box plots.

The results of the GWAS from MLM are presented in the Manhattan plots and the corresponding Quantile-Quantile plots (QQ-plots) are displayed to compare the quantiles of the empirical distribution of the results obtained in this study with those of the distribution that we would expect theoretically if the null hypothesis is true.

## 5. Conclusions

Our research identified new sources of resistance to tan spot in CIMMYT’s SHW that can be used in wheat breeding via crosses and backcrosses with elite bread wheat lines. A total of 30 significant marker-trait associations were found on chromosomes 1B, 1D, 2A, 2D, 3A, 3D, 4B, 4D, 5A, 6A, 6B, and 7D, of which some SNP markers clustered and likely represent single QTL. Several marker-trait associations found in this study can contribute to the genetic diversity of resistance, specifically those on D genome contributed by *Ae. tauschii*, which were almost all novel, but also several on the A- and B-genomes. Furthermore, our study supports the previous concept of possible inter-locus effects caused by the activation of resistance genes in the DW genomes by interaction with the D genome of *Ae. tauschii* after hybridization.

## Figures and Tables

**Table 1:** Reaction to tan spot in 40 durum wheat (DW) parents and their respective synthetic hexaploid wheat (SHW) progeny groups. Reactions are defined as Resistant (R, 1.0–1.5), Moderately Resistant (MR, 1.6–2.5), Moderately Susceptible (MS, 2.6–3.5), and Susceptible (S, 3.6–5.0).

	DW Parents			SHW	
Pedigree	Tan Spot Scores	Reaction Type	Number of SHW Progeny	Mean Tan Spot Scores	Mean Reaction Type
BOTNO	4.3	S	1	2.2	MR
SCAUP	3.9	S	3	2.2	MR
CROC_1	3.7	S	30	1.7	MR
D67.2/PARANA 66.270	3.7	S	13	1.7	MR
YAR	3.7	S	4	1.4	R
68.111/RGB-U//WARD RESEL/3/STIL	3.6	S	31	1.5	R
DECOY 1	3.5	MS	30	2.1	MR
SORA	3.4	MS	14	1.6	MR
6973/WARD.7463//74110	3.3	MS	3	1.6	MR
CPI8/GEDIZ/3/GOO//ALB/CRA	3.3	MS	31	1.9	MR
LCK59.61	3.2	MS	2	2.3	MR
68.111/RGB-U//WARD	3.1	MS	7	1.6	MR
CHEN_7	3.0	MS	1	1.2	R
ALG86/4/FGO/PALES//MEXI_1/3/RUFF/FGO/5/ENTE	2.9	MS	3	2	MR
YAV_2/TEZ	2.9	MS	12	1.6	MR
LOCAL RED	2.9	MS	7	2.2	MR
TK SN1081	2.9	MS	3	1.2	R
YARMUK	2.8	MS	4	1.7	MR
ROK/KML	2.7	MS	4	2.2	MR
STY,DR/CELTA//PALS/3/SRN_5	2.7	MS	2	1.5	R
ALTAR 84	2.6	MS	20	1.6	MR
ACONCHI 89	2.6	MS	4	1.5	R
DVERD_2	2.5	MR	13	1.5	R
FGO/USA2111	2.5	MR	1	1.1	R
ARLIN_1	2.4	MR	13	1.5	R
68.111/RGB-U//WARD/3/FGO/4/RABI	2.4	MR	31	1.5	R
SCOT/MEXI_1	2.4	MR	1	1.8	MR
GARZA/BOY	2.3	MR	7	1.8	MR
68112/WARD	2.3	MR	4	1.2	R
LARU	2.3	MR	4	1.1	R
RASCON_37	2.2	MR	2	1.3	R
KAPUDE_1	2.1	MR	1	1.9	MR
CERCETA	1.9	MR	54	1.6	MR
RABI//GS/CRA	1.6	MR	4	1.5	R
SNIPE/YAV79//DACK/TEAL	1.5	R	7	1.1	R
FALCIN_1	1.5	R	5	1.9	MR
SHAG_22	1.5	R	6	1.5	R
GREEN_3	1.2	R	1	1	R
GAN	1.1	R	39	1.4	R
SCOOP_1	1.1	R	3	1	R
Erik (R check)	1.0	R	---	---	---
Glenlea (S check)	4.8	S	---	---	---
6B-662 (MR check)	2.0	MR	---	---	---
6B-365 (MS check)	3.1	MS	---	---	---

**Table 2:** Significant markers associated with seedling resistance to tan spot detected with the consensus genetic maps. Allele ID, genetic position in centimorgan (cM), F statistics, Probability (Prob), Marker R^2^, −log10 *p*-value and the effect of allele substitution are given for each marker.

Chr	Marker ID	Allele ID	Genetic Position on Consensus Map (cM)	F Statistics	Prob.	Marker R^2^	−log10 *p*-Value	Effect of Allele Substitution (Genotype Effect)
1B	987556	987556|F|0-61:G>A-61:G>A	60.43	8.36	2.78 × 10^−4^	0.042	3.56	−0.22
1B	6045377	6045377|F|0-16:T>C-16:T>C	51.29	8.06	3.71 × 10^−4^	0.040	3.43	−0.10
1B	1089962	1089962|F|0-56:C>T-56:C>T	83.57	7.21	8.40 × 10^−4^	0.036	3.08	−0.19
2A	1070935	1070935|F|0-45:G>A-45:G>A	68.84	7.48	6.46 × 10^−4^	0.038	3.19	−0.28
4A	4993454	4993454|F|0-12:T>C-12:T>C	10.72	8.20	3.24 × 10^−4^	0.041	3.49	−0.55
5A	1200982	1200982|F|0-30:C>G-30:C>G	47.79	7.68	5.36 × 10^−4^	0.038	3.27	0.05
5A	4393896	4393896|F|0-34:T>C-34:T>C	48.67	7.21	8.43 × 10^−4^	0.036	3.07	−0.20
5B	100034112	100034112|F|0-10:C>T-10:C>T	39.26	7.80	4.77 × 10^−4^	0.039	3.32	−0.14
6A	1862737	1862737|F|0-44:C>G-44:C>G	90.36	9.15	1.30 × 10^−4^	0.046	3.89	−0.20
6A	100027398	100027398|F|0-42:A>G-42:A>G	77.32	8.21	3.20 × 10^−4^	0.041	3.49	−0.15
6A	5331622	5331622|F|0-5:A>G-5:A>G	98.51	8.05	3.72 × 10^−4^	0.040	3.43	−0.12
6A	1254459	1254459|F|0-8:A>C-8:A>C	94.09	7.35	7.36 × 10^−4^	0.037	3.13	−0.22
6A	4993056	4993056|F|0-26:A>T-26:A>T	91.17	7.18	8.68 × 10^−4^	0.036	3.06	−0.23
6B	1019955	1019955|F|0-55:A>G-55:A>G	46.69	8.82	1.79 × 10^−4^	0.044	3.75	−0.44
7D	991140	991140|F|0-11:G>C-11:G>C	153.02	10.19	4.84 × 10^−5^	0.051	4.31	−0.15
7D	993425	993425|F|0-28:A>G-28:A>G	168.74	8.35	2.81 × 10^−4^	0.041	3.55	0.59

**Table 3:** Significant markers for seedling resistance to tan spot detected with the physical map based on the Chinese spring reference genome (RefSeqV.1.0). Allele ID, physical position in CS, F statistics, Probability (Prob), Marker R^2^, −log10 *p*-value and the effect of allele substitution are given for each marker.

Chr	Marker	Allele ID	Pos	F Statistic	Prob.	Marker R^2^	−log10 *p*-Value	Effect of Allele Substitution (Genotype Effect)
1B	1089962	1089962|F|0-56:C>T-56:C>T	340462174	7.37	7.23 × 10^−4^	0.037	3.14	−0.19
1D	3026113	3026113|F|0-19:G>T-19:G>T	375647840	7.92	4.22 × 10^−4^	0.040	3.37	0.16
2A	1070935	1070935|F|0-45:G>A-45:G>A	525822786	7.99	3.97 × 10^−4^	0.040	3.40	−0.29
3A	1019955	1019955|F|0-55:A>G-55:A>G	474447292	9.28	1.16 × 10^−4^	0.046	3.94	−0.44
3A	1668224	1668224|F|0-18:T>C-18:T>C	468520788	7.03	1.00 × 10^−3^	0.035	3.00	−0.24
3D	1125862	1125862|F|0-8:C>A-8:C>A	603632716	8.86	1.72 × 10^−4^	0.044	3.76	−0.13
3D	1217411	1217411|F|0-6:C>T-6:C>T	610566593	8.06	3.71 × 10^−4^	0.040	3.43	−0.21
3D	987556	987556|F|0-61:G>A-61:G>A	288544777	7.88	4.41 × 10^−4^	0.039	3.36	−0.21
4D	4993454	4993454|F|0-12:T>C-12:T>C	449396486	8.57	2.26 × 10^−4^	0.043	3.64	−0.56
5A	100034112	100034112|F|0-10:C>T-10:C>T	471723681	8.21	3.20 × 10^−4^	0.041	3.50	−0.15
5A	1200982	1200982|F|0-30:C>G-30:C>G	454770585	7.28	7.83 × 10^−4^	0.036	3.11	−0.05
6A	100027398	100027398|F|0-42:A>G-42:A>G	601233092	8.92	1.62 × 10^−4^	0.045	3.79	−0.15
6A	1254459	1254459|F|0-8:A>C-8:A>C	602989232	8.23	3.15 × 10^−4^	0.041	3.50	−0.23
6A	2266481	2266481|F|0-54:C>T-54:C>T	602745555	7.19	8.56 × 10^−4^	0.036	3.07	−0.21
6B	1862737	1862737|F|0-44:C>G-44:C>G	689032602	9.46	9.65 × 10^−5^	0.047	4.02	−0.20
6B	1112961	1112961|F|0-43:G>A-43:G>A	62173247	7.44	6.75 × 10^−4^	0.037	3.17	−0.13
7D	16793126	16793126|F|0-15:G>T-15:G>T	161842641	9.59	8.59 × 10^−5^	0.048	4.07	0.05
7D	993425	993425|F|0-28:A>G-28:A>G	620252466	8.28	3.00 × 10^−4^	0.041	3.52	0.61

**Table 4:** Significant markers associated with seedling resistance to tan spot based on durum wheat (cv. Svevo) and *Ae. tauschii* reference genomes. Allele ID, physical positions, F-statistics, Probability (Prob), Marker R^2^, log10 *p*-value and the effect of allele substitution are given for each marker.

Chr	Marker	Allelle ID	Position	F Statistic	Prob.	Marker R^2^	−log10 *p*-Value	Effect of Allele Substitution (Genotype Effect)
1B	1106306	1106306|F|0-31:A>G-31:A>G	18733634	9.04	1.45 × 10^−4^	0.045	3.84	−0.24
1B	1089962	1089962|F|0-56:C>T-56:C>T	333205076	8.01	3.89 × 10^−4^	0.040	3.41	−0.20
1B	3026113	3026113|F|0-19:G>T-19:G>T	493514948	7.86	4.47 × 10^−4^	0.039	3.35	0.16
1B	4909460	4909460|F|0-15:T>C-15:T>C	551136407	7.33	7.45 × 10^−4^	0.037	3.13	−0.17
2D	1046601	1046601|F|0-37:C>G-37:C>G	543349511	7.33	7.47 × 10^−4^	0.037	3.13	−0.01
2D	1217245	1217245|F|0-50:G>A-50:G>A	49063764	7.27	7.90 × 10^−4^	0.036	3.10	−0.15
3A	1065211	1065211|F|0-46:G>A-46:G>A	477078596	7.49	6.43 × 10^−4^	0.037	3.19	−0.26
3A	1125872	1125872|F|0-29:C>T-29:C>T	141341740	7.14	9.00 × 10^−4^	0.036	3.05	−0.27
4A	1125862	1125862|F|0-8:C>A-8:C>A	558758715	8.35	2.80 × 10^−4^	0.042	3.55	−0.14
5A	3064590	3064590|F|0-39:T>A-39:T>A	433029624	7.17	8.76 × 10^−4^	0.036	3.06	−0.22
6A	100027398	100027398|F|0-42:A>G-42:A>G	597038442	11.53	1.36 × 10^−5^	0.058	4.87	−0.17
6A	1254459	1254459|F|0-8:A>C-8:A>C	598610204	8.69	2.01 × 10^−4^	0.043	3.70	−0.23
7D	16793126	16793126|F|0-15:G>T-15:G>T	162738314	9.30	1.13 × 10^−4^	0.047	3.95	0.05

**Table 5:** List of potential candidate genes found in regions identified by marker-trait associations for seedling resistance to tan spot based on Consensus Map, Physical Map (Chinese spring Ref Seq_v1.0) and Durum Wheat (cv. Svevo) aligned to *Ae. tauschii*. Information on chromosome (Ch.), marker, genetic position on the consensus map (cM), position on the Chinese Spring RefV.10, gene ID (CS), GWAS, *p*-value, marker R2 and −log10 *p*-value is given. Underlined marker ID, Consensus map, and Position (CS) indicate candidate genes.

Ch.	Marker ID	Consensus Map (cM)	Position (CS)	Position (Svevo)	Pos (Ae.t.)	Gene (s)	GWAS	*p*-Value	Marker R^2^	−log10 *p*-Value
1B	1106306			1B-18733634		-	Durum-*tauschii* (phy. pos)	1.45 × 10^−4^	0.045	3.84
1B	6045377	1B-51.3					Bread wheat (genetic map)	3.71 × 10^−4^	0.040	3.43
1B	1089962	1B-83.6	1B-340462174	1B-333205076			*Aestivum* (genetic map)	8.40 × 10^−4^	0.036	3.08
						-	*Aestivum* (phy. pos.)	7.23 × 10^−4^	0.037	3.14
						-	Durum-*tauschii* (phy. pos)	3.89 × 10^−4^	0.040	3.41
1B	4909460		1B-558561647	1B-551136407		-	Durum-*tauschii* (phy. pos)	7.45 × 10^−4^	0.037	3.13
1D	3026113		1D-375647840		1D-381593800	-	*Aestivum* (phy. pos.)	4.22 × 10^−4^	0.040	3.37
						AET1Gv20669700	Durum-*tauschii* (phy. pos)	4.47 × 10^−4^	0.039	3.35
2A	1070935	2A-68.8					*Aestivum* (genetic map)	6.46 × 10^−4^	0.038	3.19
			2A-525822786	2A-519747584		-	Aestivum (phy. pos.)	3.97 × 10^−4^	0.040	3.40
2D	1217245		2D-48123061		2D-49063764	-	Durum-*tauschii* (phy. pos)	7.90 × 10^−4^	0.036	3.10
2D	1046601		2D-544685083		2D-543349511	TraesCS2D02G432700	Durum-*tauschii* (phy. pos)	7.47 × 10^−4^	0.037	3.13
3A	1125872		3A-135590641	3A-141341769		-	Durum-*tauschii* (phy. pos)	9.00 × 10^−4^	0.036	3.05
3A	1668224		3A-468520788	3A-471432162		-	*Aestivum* (phy. pos.)	1.00 × 10^−3^	0.035	3.00
3A or 6B	1019955	6B-46.7	3A-474447292, 6B-665557108	3A-477078694			*Aestivum* (genetic map)	1.79 × 10^−4^	0.044	3.75
						-	*Aestivum* (phy. pos.)	1.16 × 10^−4^	0.046	3.94
3A	1065211		3A-474447226	3A-477078596		-	Durum-*tauschii* (phy. pos)	6.43 × 10^−4^	0.037	3.19
3D	987556	1B-60.4					*Aestivum* (genetic map)	2.78 × 10^−4^	0.042	3.56
			3D-288544838		3D-295969303	-	*Aestivum* (phy. pos.)	4.41 × 10^−4^	0.039	3.36
3D	1125862		3D-603632716		3D-614682837	-	*Aestivum* (phy. pos.)	1.72 × 10^−4^	0.044	3.76
						-	Durum-*tauschii* (phy. pos)	2.80 × 10^−4^	0.042	3.55
3D	1217411		3D-610566592		3D-622597928	-	*Aestivum* (phy. pos.)	3.71 × 10^−4^	0.040	3.43
4B or 4D	4993454	4A-10.7	4B-561892901, 4D-449396542	4B-566325530	4D-455660733		*Aestivum* (genetic map)	3.24 × 10^−4^	0.041	3.49
						-	*Aestivum* (phy. pos.)	2.26 × 10^−4^	0.043	3.64
5A	4393896	5A-48.7					*Aestivum* (genetic map)	8.43 × 10^−4^	0.036	3.07
5A	1200982	5A-47.8	5A-454770615	5A-416482338			*Aestivum* (genetic map)	5.36 × 10^−4^	0.038	3.27
						TraesCS5A02G238600 TRITD5Av1G148960	*Aestivum* (phy. pos.)	7.83 × 10^−4^	0.036	3.11
5A	100034112	5B-39.3	5A-471723681	5A-433814227			*Aestivum* (genetic map)	4.77 × 10^−4^	0.039	3.32
						-	*Aestivum* (phy. pos.)	3.20 × 10^−4^	0.041	3.50
5A	3064590		5A-470186523	5A:433029663		TraesCS5A02G254500 TRITD5Av1G155700	Durum-*tauschii* (phy. pos)	8.76 × 10^−4^	0.036	3.06
6A	1862737	6A-90.4	6A-599622814	6A-595687891			*Aestivum* (genetic map)	1.30 × 10^−4^	0.046	3.89
						TraesCS6A02G378800, TRITD6Av1G217060	*Aestivum* (phy. pos.)	9.65 × 10^−5^	0.047	4.02
6A	100027398	6A-77.3	6A-601233092	6A-597038469			*Aestivum* (genetic map)	3.20 × 10^−4^	0.041	3.49
						TraesCS6A02G381900	*Aestivum* (phy. pos.)	1.62 × 10^−4^	0.045	3.79
						TRITD6Av1G217800	Durum-*tauschii* (phy. pos)	1.36 × 10^−5^	0.058	4.87
6A	1254459	6A-94.1	6A-602989232	6A-598610265			*Aestivum* (genetic map)	7.36 × 10^−4^	0.037	3.13
						-	*Aestivum* (phy. pos.)	3.15 × 10^−4^	0.041	3.50
						-	Durum-*tauschii* (phy. pos)	2.01 × 10^−4^	0.043	3.70
6A	2266481		6A-602745555	6A-598380242		TraesCS6A02G384200	*Aestivum* (phy. pos.)	8.56 × 10^−4^	0.036	3.07
6A	4993056	6A-91.2					*Aestivum* (genetic map)	8.68 × 10^−4^	0.036	3.06
6A	5331622	6A-98.6					*Aestivum* (genetic map)	3.72 × 10^−4^	0.040	3.43
6B	1112961		6B-62173280	6B-59030547		-	*Aestivum* (phy. pos.)	6.75 × 10^−4^	0.037	3.17
7D	16793126		7D-161842695		7D-162738368	TraesCS7D02G203900	*Aestivum* (phy. pos.)	8.59 × 10^−5^	0.048	4.07
						AET7Gv20511100 AET7Gv20511200	Durum-*tauschii* (phy. pos)	1.13 × 10^−4^	0.047	3.95
7D	991140	7D-153.0					*Aestivum* (genetic map)	4.84 × 10^−5^	0.051	4.31
7D	993425	7D-168.7	7D-620252508		7D-625050620		*Aestivum* (genetic map)	2.81 × 10^−4^	0.042	3.55
						TraesCS7D02G524200 AET7Gv21298500	*Aestivum* (phy. pos.)	3.00 × 10^−4^	0.041	3.52

## Data Availability

The original contributions presented in the study are publicly available on Supplementary Tables S1 and S2 and Figure S1.

## Supplementary Materials

The following supporting information can be downloaded at: https://www.mdpi.com/article/10.3390/plants11030433/s1, Figure S1: Principal component analysis of the synthetic wheat panel used in this study; Table S1: Seedling tan spot reaction scores of synthetic hexaploid wheat (SHW) lines and their durum wheat (DW) parents, Table S2: Candidate genes for significant marker-trait associations identified from *Triticum aestivum* (IWGSC), *Triticum turgidum* (Svevo.v1), *Aegilops tauschii* (Aet_v4.0), and *Triticum dicoccoides* (WEWSeq_v.1.0).
