# Supplementary material for: Genome-Wide Association Study for Resistance to Tan Spot in Synthetic Hexaploid Wheat
Source: Plants (Basel). 2022 Feb 5;11(3):433. doi: 10.3390/plants11030433 (PMC8839754; doi:10.3390/plants11030433)

Supplementary Table S1. Seedling tan spot reaction scores of synthetic hexaploid wheat (SHW) lines and their durum wheat (DW) parents

		Reaction to PTR		
Entry No.	Pedigree	AVG**	Score	Number of progeny
1	**BOTNO***	4.31	S	1
2	BOTNO/AE.SQUARROSA (617)	2.2	MR	--
3	**68.111/RGB-U//WARD RESEL/3/STIL***	3.56	S	31
4	68.111/RGB-U//WARD RESEL/3/STIL/4/AE.SQUARROSA (332)	1.00	R	--
5	68.111/RGB-U//WARD RESEL/3/STIL/4/AE.SQUARROSA (1030)	1.08	R	--
6	68.111/RGB-U//WARD RESEL/3/STIL/4/AE.SQUARROSA (389)	1.14	R	--
7	68.111/RGB-U//WARD RESEL/3/STIL/4/AE.SQUARROSA (630)	1.16	R	--
8	68.111/RGB-U//WARD RESEL/3/STIL/4/AE.SQUARROSA (628)	1.17	R	--
9	68.111/RGB-U//WARD RESEL/3/STIL/4/AE.SQUARROSA (392)	1.19	R	--
10	68.111/RGB-U//WARD RESEL/3/STIL/4/AE.SQUARROSA (684)	1.21	R	--
11	68.111/RGB-U//WARD RESEL/3/STIL/4/AE.SQUARROSA (631)	1.22	R	--
12	68.111/RGB-U//WARD RESEL/3/STIL/4/AE.SQUARROSA (631)	1.23	R	--
13	68.111/RGB-U//WARD RESEL/3/STIL/4/AE.SQUARROSA (627)	1.23	R	--
14	68.111/RGB-U//WARD RESEL/3/STIL/4/AE.SQUARROSA (659)	1.24	R	--
15	68.111/RGB-U//WARD RESEL/3/STIL/4/AE.SQUARROSA (390)	1.25	R	--
16	68.111/RGB-U//WARD RESEL/3/STIL/4/AE.SQUARROSA (623)	1.28	R	--
17	68.111/RGB-U//WARD RESEL/3/STIL/4/AE.SQUARROSA (1038)	1.30	R	--
18	68.111/RGB-U//WARD RESEL/3/STIL/4/AE.SQUARROSA (781)	1.40	R	--
19	68.111/RGB-U//WARD RESEL/3/STIL/4/AE.SQUARROSA (1029)	1.41	R	--
20	68.111/RGB-U//WARD RESEL/3/STIL/4/AE.SQUARROSA (675)	1.41	R	--
21	68.111/RGB-U//WARD RESEL/3/STIL/4/AE.SQUARROSA (385)	1.45	R	--
22	68.111/RGB-U//WARD RESEL/3/STIL/4/AE.SQUARROSA (386)	1.50	R	--
23	68.111/RGB-U//WARD RESEL/3/STIL/4/AE.SQUARROSA (625)	1.51	R	--

24	68.111/RGB-U//WARD RESEL/3/STIL/4/AE.SQUARROSA (768)	1.54	R	--
25	68.111/RGB-U//WARD RESEL/3/STIL/4/AE.SQUARROSA (783)	1.64	R	--
26	68.111/RGB-U//WARD RESEL/3/STIL/4/AE.SQUARROSA (164)	1.75	R	--
27	68.111/RGB-U//WARD RESEL/3/STIL/4/AE.SQUARROSA (672)	1.83	MR	--
28	68.111/RGB-U//WARD RESEL/3/STIL/4/AE.SQUARROSA (681)	1.84	MR	--
29	68.111/RGB-U//WARD RESEL/3/STIL/4/AE.SQUARROSA (685)	1.85	MR	--
30	68.111/RGB-U//WARD RESEL/3/STIL/4/AE.SQUARROSA (700)	1.88	MR	--
31	68.111/RGB-U//WARD RESEL/3/STIL/4/AE.SQUARROSA (662)	1.92	MR	--
32	68.111/RGB-U//WARD RESEL/3/STIL/4/AE.SQUARROSA (1090)	2.05	MR	--
33	68.111/RGB-U//WARD RESEL/3/STIL/4/AE.SQUARROSA (188)	2.29	MR	--
34	68.111/RGB-U//WARD RESEL/3/STIL/4/AE.SQUARROSA (1010)	2.68	MS	--
35	**68.111/RGB-U//WARD***	3.15	MS	7
36	68.111/RGB-U//WARD/3/AE.SQUARROSA (202)	1.75	MR	--
37	68.111/RGB-U//WARD/3/AE.SQUARROSA (426)	1.00	R	--
38	68.111/RGB-U//WARD/3/AE.SQUARROSA (316)	1.39	R	--
39	68.111/RGB-U//WARD/3/AE.SQUARROSA (329)	1.43	R	--
40	68.111/RGB-U//WARD/3/AE.SQUARROSA (322)	1.50	R	--
41	68.111/RGB-U//WARD/3/AE.SQUARROSA (321)	1.73	MR	--
42	68.111/RGB-U//WARD/3/AE.SQUARROSA (511)	2.09	MR	--
43	**68.111/RGB-U//WARD/3/FGO/4/RABI***	2.41	MR	31
44	68.111/RGB-U//WARD/3/FGO/4/RABI/5/AE.SQUARROSA (882)	1.03	R	--
45	68.111/RGB-U//WARD/3/FGO/4/RABI/5/AE.SQUARROSA (878)	1.08	R	--
46	68.111/RGB-U//WARD/3/FGO/4/RABI/5/AE.SQUARROSA (701)	1.08	R	--
47	68.111/RGB-U//WARD/3/FGO/4/RABI/5/AE.SQUARROSA (1050)	1.08	R	--
48	68.111/RGB-U//WARD/3/FGO/4/RABI/5/AE.SQUARROSA (675)	1.08	R	--
49	68.111/RGB-U//WARD/3/FGO/4/RABI/5/AE.SQUARROSA (778)	1.08	R	--
50	68.111/RGB-U//WARD/3/FGO/4/RABI/5/AE.SQUARROSA (768)	1.10	R	--
51	68.111/RGB-U//WARD/3/FGO/4/RABI/5/AE.SQUARROSA (720)	1.13	R	--
52	68.111/RGB-U//WARD/3/FGO/4/RABI/5/AE.SQUARROSA (809)	1.20	R	--
53	68.111/RGB-U//WARD/3/FGO/4/RABI/5/AE.SQUARROSA (710)	1.21	R	--

54	68.111/RGB-U//WARD/3/FGO/4/RABI/5/AE.SQUARROSA (661)	1.27	R	--
55	68.111/RGB-U//WARD/3/FGO/4/RABI/5/AE.SQUARROSA (191)	1.28	R	--
56	68.111/RGB-U//WARD/3/FGO/4/RABI/5/AE.SQUARROSA (878)	1.29	R	--
57	68.111/RGB-U//WARD/3/FGO/4/RABI/5/AE.SQUARROSA (719)	1.30	R	--
58	68.111/RGB-U//WARD/3/FGO/4/RABI/5/AE.SQUARROSA (974)	1.30	R	--
59	68.111/RGB-U//WARD/3/FGO/4/RABI/5/AE.SQUARROSA (905)	1.30	R	--
60	68.111/RGB-U//WARD/3/FGO/4/RABI/5/AE.SQUARROSA (1093)	1.33	R	--
61	68.111/RGB-U//WARD/3/FGO/4/RABI/5/AE.SQUARROSA (788)	1.35	R	--
62	68.111/RGB-U//WARD/3/FGO/4/RABI/5/AE.SQUARROSA (878)	1.42	R	--
63	68.111/RGB-U//WARD/3/FGO/4/RABI/5/AE.SQUARROSA (504)	1.43	R	--
64	68.111/RGB-U//WARD/3/FGO/4/RABI/5/AE.SQUARROSA (809)	1.50	R	--
65	68.111/RGB-U//WARD/3/FGO/4/RABI/5/AE.SQUARROSA (878)	1.58	MR	--
66	68.111/RGB-U//WARD/3/FGO/4/RABI/5/AE.SQUARROSA (784)	1.59	MR	--
67	68.111/RGB-U//WARD/3/FGO/4/RABI/5/AE.SQUARROSA (878)	1.60	MR	--
68	68.111/RGB-U//WARD/3/FGO/4/RABI/5/AE.SQUARROSA (878)	1.67	MR	--
69	68.111/RGB-U//WARD/3/FGO/4/RABI/5/AE.SQUARROSA (709)	1.84	MR	--
70	68.111/RGB-U//WARD/3/FGO/4/RABI/5/AE.SQUARROSA (878)	1.91	MR	--
71	68.111/RGB-U//WARD/3/FGO/4/RABI/5/AE.SQUARROSA (809)	1.92	MR	--
72	68.111/RGB-U//WARD/3/FGO/4/RABI/5/AE.SQUARROSA (1010)	1.99	MR	--
73	68.111/RGB-U//WARD/3/FGO/4/RABI/5/AE.SQUARROSA (878)	2.42	MR	--
74	68.111/RGB-U//WARD/3/FGO/4/RABI/5/AE.SQUARROSA (878)	2.61	MS	--
75	**68112/WARD***	2.34	MR	4
76	68112/WARD//AE.SQUARROSA (369)	1.05	R	
77	68112/WARD//AE.SQUARROSA (369)	1.15	R	
78	68112/WARD//AE.SQUARROSA (369)	1.21	R	
79	68112/WARD//AE.SQUARROSA (369)	1.22	R	
80	**6973/WARD.7463//74110***	3.34	MS	3
81	6973/WARD.7463//74110/3/AE.SQUARROSA (665)	1.00	R	
82	6973/WARD.7463//74110/3/AE.SQUARROSA (438)	1.11	R	
83	6973/WARD.7463//74110/3/AE.SQUARROSA (35A)	2.63	MS	

84	**ACONCHI 89***	2.55	MS	4
85	ACO89/AE.SQUARROSA (178)	1.50	R	
86	ACO89/AE.SQUARROSA (309)	1.08	R	
87	ACO89/AE.SQUARROSA (290)	1.48	R	
88	ACO89/AE.SQUARROSA (282)	2.11	MR	
89	**ALG86/4/FGO/PALES//MEXI_1/3/RUFF/FGO/5/ENTE***	2.94	MS	3
90	ALG86/4/FGO/PALES//MEXI_1/3/RUFF/FGO/5/ENTE/6/AE.SQUARROSA (389)	2.08	MR	
91	ALG86/4/FGO/PALES//MEXI_1/3/RUFF/FGO/5/ENTE/6/AE.SQUARROSA (451)	2.31	MR	
92	ALG86/4/FGO/PALES//MEXI_1/3/RUFF/FGO/5/ENTE/6/AE.SQUARROSA (723)	1.73	MR	
93	**ALTAR 84***	2.59	MS	20
94	ALTAR 84/AE.SQUARROSA (1012)	2.06	MR	
95	ALTAR 84/AE.SQUARROSA (174)	2.05	MR	
96	ALTAR 84/AE.SQUARROSA (188)	1.12	R	
97	ALTAR 84/AE.SQUARROSA (191)	1.06	R	
98	ALTAR 84/AE.SQUARROSA (198)	1.89	R	
99	ALTAR 84/AE.SQUARROSA (220)	1.44	R	
100	ALTAR 84/AE.SQUARROSA (221)	1.11	R	
101	ALTAR 84/AE.SQUARROSA (223)	1.39	R	
102	ALTAR 84/AE.SQUARROSA (224)	1.37	R	
103	ALTAR 84/AE.SQUARROSA (224)	1.22	R	
104	ALTAR 84/AE.SQUARROSA (224)	1.04	R	
105	ALTAR 84/AE.SQUARROSA (244)	1.69	MR	
106	ALTAR 84/AE.SQUARROSA (291)	1.34	R	
107	ALTAR 84/AE.SQUARROSA (319)	1.45	R	
108	ALTAR 84/AE.SQUARROSA (333)	1.94	MR	
109	ALTAR 84/AE.SQUARROSA (507)	2.19	MR	
110	ALTAR 84/AE.SQUARROSA (531)	1.52	R	
111	ALTAR 84/AE.SQUARROSA (539)	2.52	MR	

112	ALTAR 84/AE.SQUARROSA (793)	1.46	R	
113	ALTAR 84/AE.SQUARROSA(Y86-87 S401)	1.28	R	
114	**ARLIN_1***	2.44	MR	13
115	AE.SQUARROSA (1031)/ARLIN_1	1.02	R	
116	ARLIN/AE.SQUARROSA (283)	2.29	MR	
117	ARLIN/AE.SQUARROSA (317)	1.16	R	
118	ARLIN/AE.SQUARROSA (410)	2.65	MS	
119	ARLIN_1/AE.SQUARROSA (1018)	1.07	R	
120	ARLIN_1/AE.SQUARROSA (310)	1.00	R	
121	ARLIN_1/AE.SQUARROSA (320)	1.00	R	
122	ARLIN_1/AE.SQUARROSA (333)	1.53	R	
123	ARLIN_1/AE.SQUARROSA (335)	1.03	R	
124	ARLIN_1/AE.SQUARROSA (368)	1.23	R	
125	ARLIN_1/AE.SQUARROSA (430)	1.06	R	
126	ARLIN_1/AE.SQUARROSA (536)	2.40	MR	
127	ARLIN_1/AE.SQUARROSA (802)	1.59	MR	
128	**CERCETA***	1.85	MR	54
129	CETA/AE.SQUAROOSA (263)	1.65	MR	--
130	CETA/AE.SQUARROSA (1016)	1.66	MR	--
131	CETA/AE.SQUARROSA (1018)	1.22	R	--
132	CETA/AE.SQUARROSA (1026)	1.20	R	--
133	CETA/AE.SQUARROSA (1027)	1.09	R	--
134	CETA/AE.SQUARROSA (1030)	1.24	R	--
135	CETA/AE.SQUARROSA (1031)	1.28	R	--
136	CETA/AE.SQUARROSA (1036)	1.73	MR	--
137	CETA/AE.SQUARROSA (1038)	1.00	R	--
138	CETA/AE.SQUARROSA (1043)	1.88	MR	--
139	CETA/AE.SQUARROSA (1047)	1.70	MR	--
140	CETA/AE.SQUARROSA (1053)	1.87	MR	--
141	CETA/AE.SQUARROSA (1073)	1.69	MR	--

142	CETA/AE.SQUARROSA (1090)	1.72	MR	--
143	CETA/AE.SQUARROSA (166)	1.13	R	--
144	CETA/AE.SQUARROSA (174)	1.47	R	--
145	CETA/AE.SQUARROSA (187)	1.22	R	--
146	CETA/AE.SQUARROSA (230)	1.05	R	--
147	CETA/AE.SQUARROSA (231)	1.79	MR	--
148	CETA/AE.SQUARROSA (244)	2.25	MR	--
149	CETA/AE.SQUARROSA (246)	2.30	MR	--
150	CETA/AE.SQUARROSA (248)	2.88	MS	--
151	CETA/AE.SQUARROSA (262)	1.33	R	--
152	CETA/AE.SQUARROSA (310)	1.03	R	--
153	CETA/AE.SQUARROSA (335)	1.09	R	--
154	CETA/AE.SQUARROSA (356)	1.86	R	--
155	CETA/AE.SQUARROSA (371)	1.17	R	--
156	CETA/AE.SQUARROSA (391)	1.16	R	--
157	CETA/AE.SQUARROSA (418)	1.08	R	--
158	CETA/AE.SQUARROSA (442)	1.35	R	--
159	CETA/AE.SQUARROSA (445)	2.30	MR	--
160	CETA/AE.SQUARROSA (450)	1.70	MR	
161	CETA/AE.SQUARROSA (485)	1.78	MR	
162	CETA/AE.SQUARROSA (496)	1.08	R	
163	CETA/AE.SQUARROSA (499)	1.52	R	
164	CETA/AE.SQUARROSA (506)	2.42	MR	
165	CETA/AE.SQUARROSA (525)	1.86	MR	
166	CETA/AE.SQUARROSA (530)	2.43	MR	
167	CETA/AE.SQUARROSA (533)	2.66	MS	
168	CETA/AE.SQUARROSA (539)	2.46	MR	
169	CETA/AE.SQUARROSA (540)	2.48	MR	
170	CETA/AE.SQUARROSA (541)	2.72	MS	
171	CETA/AE.SQUARROSA (615)	1.52	R	

172	CETA/AE.SQUARROSA (629)	1.74	MR	
173	CETA/AE.SQUARROSA (681)	1.35	R	
174	CETA/AE.SQUARROSA (682)	1.52	R	
175	CETA/AE.SQUARROSA (683)	1.51	R	
176	CETA/AE.SQUARROSA (684)	1.27	R	
177	CETA/AE.SQUARROSA (750)	1.68	MR	
178	CETA/AE.SQUARROSA (783)	1.23	R	
179	CETA/AE.SQUARROSA (796)	1.53	R	
180	CETA/AE.SQUARROSA (895)	1.35	R	
181	CETA/AE.SQUARROSA (895)	1.11	R	
182	CETA/T.URARTU (557)	1.05	R	
183	**CHEN_7***	3.01	MS	1
184	CHEN_7/AE.SQUARROSA (429)	1.19	R	
185	**CPI8/GEDIZ/3/GOO//ALB/CRA***	3.25	MS	31
186	CPI8/GEDIZ/3/GOO//ALB/CRA/4/AE.SQUARROSA (1017)	1.95	MR	
187	CPI8/GEDIZ/3/GOO//ALB/CRA/4/AE.SQUARROSA (1018)	1.69	MR	
188	CPI8/GEDIZ/3/GOO//ALB/CRA/4/AE.SQUARROSA (1021)	1.78	MR	
189	CPI8/GEDIZ/3/GOO//ALB/CRA/4/AE.SQUARROSA (1026)	1.86	MR	
190	CPI8/GEDIZ/3/GOO//ALB/CRA/4/AE.SQUARROSA (1029)	1.69	MR	
191	CPI8/GEDIZ/3/GOO//ALB/CRA/4/AE.SQUARROSA (1031)	1.83	MR	
192	CPI8/GEDIZ/3/GOO//ALB/CRA/4/AE.SQUARROSA (184)	1.69	MR	
193	CPI8/GEDIZ/3/GOO//ALB/CRA/4/AE.SQUARROSA (188)	2.49	MR	
194	CPI8/GEDIZ/3/GOO//ALB/CRA/4/AE.SQUARROSA (193)	2.60	MS	
195	CPI8/GEDIZ/3/GOO//ALB/CRA/4/AE.SQUARROSA (196)	1.81	MR	
196	CPI8/GEDIZ/3/GOO//ALB/CRA/4/AE.SQUARROSA (205)	2.14	MR	
197	CPI8/GEDIZ/3/GOO//ALB/CRA/4/AE.SQUARROSA (208)	2.17	MR	
198	CPI8/GEDIZ/3/GOO//ALB/CRA/4/AE.SQUARROSA (215)	1.64	MR	
199	CPI8/GEDIZ/3/GOO//ALB/CRA/4/AE.SQUARROSA (227)	2.03	MR	
200	CPI8/GEDIZ/3/GOO//ALB/CRA/4/AE.SQUARROSA (244)	2.33	MR	
201	CPI8/GEDIZ/3/GOO//ALB/CRA/4/AE.SQUARROSA (273)	2.42	MR	

202	CPI8/GEDIZ/3/GOO//ALB/CRA/4/AE.SQUARROSA (296)	2.18	MR	
203	CPI8/GEDIZ/3/GOO//ALB/CRA/4/AE.SQUARROSA (305)	1.64	R	
204	CPI8/GEDIZ/3/GOO//ALB/CRA/4/AE.SQUARROSA (334)	2.09	MR	
205	CPI8/GEDIZ/3/GOO//ALB/CRA/4/AE.SQUARROSA (358)	1.35	R	
206	CPI8/GEDIZ/3/GOO//ALB/CRA/4/AE.SQUARROSA (409)	1.90	MR	
207	CPI8/GEDIZ/3/GOO//ALB/CRA/4/AE.SQUARROSA (439)	2.15	MR	
208	CPI8/GEDIZ/3/GOO//ALB/CRA/4/AE.SQUARROSA (461)	1.56	R	
209	CPI8/GEDIZ/3/GOO//ALB/CRA/4/AE.SQUARROSA (533)	2.67	MS	
210	CPI8/GEDIZ/3/GOO//ALB/CRA/4/AE.SQUARROSA (629)	1.75	MR	
211	CPI8/GEDIZ/3/GOO//ALB/CRA/4/AE.SQUARROSA (633)	1.70	MR	
212	CPI8/GEDIZ/3/GOO//ALB/CRA/4/AE.SQUARROSA (637)	2.06	MR	
213	CPI8/GEDIZ/3/GOO//ALB/CRA/4/AE.SQUARROSA (659)	1.52	R	
214	CPI8/GEDIZ/3/GOO//ALB/CRA/4/AE.SQUARROSA (684)	1.02	R	
215	CPI8/GEDIZ/3/GOO//ALB/CRA/4/AE.SQUARROSA (698)	1.98	MR	
216	AE.SQUARROSA (1043)/4/CPI8/GEDIZ/3/GOO//ALB/CRA	1.08	R	
217	**CROC_1***	3.74	S	30
218	CROC_1/AE.SQUARROSA (168)	2.33	MR	
219	CROC_1/AE.SQUARROSA (170)	2.11	MR	
220	CROC_1/AE.SQUARROSA (176)	1.63	R	
221	CROC_1/AE.SQUARROSA (177)	2.86	MS	
222	CROC_1/AE.SQUARROSA (205)	1.60	MR	
223	CROC_1/AE.SQUARROSA (210)	1.74	MR	
224	CROC_1/AE.SQUARROSA (210)	1.67	MR	
225	CROC_1/AE.SQUARROSA (210)	1.65	MR	
226	CROC_1/AE.SQUARROSA (213)	1.46	R	
227	CROC_1/AE.SQUARROSA (215)	1.92	MR	
228	CROC_1/AE.SQUARROSA (224)	1.75	MR	
229	CROC_1/AE.SQUARROSA (224)	1.25	R	
230	CROC_1/AE.SQUARROSA (224)	1.15	R	
231	CROC_1/AE.SQUARROSA (224)	1.08	R	

232	CROC_1/AE.SQUARROSA (229)	2.39	MR	
233	CROC_1/AE.SQUARROSA (239)	1.10	R	
234	CROC_1/AE.SQUARROSA (256)	2.98	MS	
235	CROC_1/AE.SQUARROSA (275)	1.77	MR	
236	CROC_1/AE.SQUARROSA (298)	2.63	MS	
237	CROC_1/AE.SQUARROSA (310)	1.33	R	
238	CROC_1/AE.SQUARROSA (333)	1.06	R	
239	CROC_1/AE.SQUARROSA (397)	1.38	R	
240	CROC_1/AE.SQUARROSA (493)	1.34	R	
241	CROC_1/AE.SQUARROSA (516)	1.08	R	
242	CROC_1/AE.SQUARROSA (517)	1.71	MR	
243	CROC_1/AE.SQUARROSA (518)	2.05	MR	
244	CROC_1/AE.SQUARROSA (662)	1.33	R	
245	CROC_1/AE.SQUARROSA (725)	1.62	MR	
246	CROC_1/AE.SQUARROSA (826)	1.44	R	
247	CROC_1/AE.SQUARROSA (886)	1.00	R	
248	**D67.2/PARANA 66.270***	3.72	S	13
249	D67.2/PARANA 66.270//AE.SQUARROSA (1148)	1.36	R	
250	D67.2/PARANA 66.270//AE.SQUARROSA (211)	1.67	R	
251	D67.2/PARANA 66.270//AE.SQUARROSA (213)	2.34	MR	
252	D67.2/PARANA 66.270//AE.SQUARROSA (218)	1.12	R	
253	D67.2/PARANA 66.270//AE.SQUARROSA (220)	1.58	MR	
254	D67.2/PARANA 66.270//AE.SQUARROSA (221)	2.00	MR	
255	D67.2/PARANA 66.270//AE.SQUARROSA (222)	2.16	MR	
256	D67.2/PARANA 66.270//AE.SQUARROSA (223)	1.55	MR	
257	D67.2/PARANA 66.270//AE.SQUARROSA (246)	2.05	MR	
258	D67.2/PARANA 66.270//AE.SQUARROSA (633)	1.91	MR	
259	D67.2/PARANA 66.270//AE.SQUARROSA (634)	1.73	MR	
260	D67.2/PARANA 66.270//AE.SQUARROSA (657)	1.91	MR	
261	D67.2/PARANA 66.270//AE.SQUARROSA (668)	1.03	R	

262	**DECOY 1***	3.50	MS	30
263	DOY1/AE.SQUARROSA (1016)	2.14	MR	
264	DOY1/AE.SQUARROSA (1018)	2.04	MR	
265	DOY1/AE.SQUARROSA (1024)	2.59	MS	
266	DOY1/AE.SQUARROSA (1026)	1.87	MR	
267	DOY1/AE.SQUARROSA (1029)	2.29	MR	
268	DOY1/AE.SQUARROSA (177)	2.56	MS	
269	DOY1/AE.SQUARROSA (188)	2.35	MR	
270	DOY1/AE.SQUARROSA (216)	1.56	MR	
271	DOY1/AE.SQUARROSA (255)	3.43	MS	
272	DOY1/AE.SQUARROSA (258)	2.29	MR	
273	DOY1/AE.SQUARROSA (267)	1.28	R	
274	DOY1/AE.SQUARROSA (295)	1.30	R	
275	DOY1/AE.SQUARROSA (322)	2.24	MR	
276	DOY1/AE.SQUARROSA (334)	1.68	MR	
277	DOY1/AE.SQUARROSA (360)	1.59	MR	
278	DOY1/AE.SQUARROSA (415)	2.41	MR	
279	DOY1/AE.SQUARROSA (428)	2.11	MR	
280	DOY1/AE.SQUARROSA (446)	1.63	MR	
281	DOY1/AE.SQUARROSA (447)	1.75	MR	
282	DOY1/AE.SQUARROSA (488)	2.06	MR	
283	DOY1/AE.SQUARROSA (507)	1.83	MR	
284	DOY1/AE.SQUARROSA (510)	1.87	MR	
285	DOY1/AE.SQUARROSA (515)	2.35	MR	
286	DOY1/AE.SQUARROSA (516)	1.43	R	
287	DOY1/AE.SQUARROSA (517)	2.78	MS	
288	DOY1/AE.SQUARROSA (532)	2.73	MS	
289	DOY1/AE.SQUARROSA (540)	2.58	MS	
290	DOY1/AE.SQUARROSA (632)	1.48	R	
291	AE.SQUARROSA (1026)/DOY1	2.51	MR	

292	AE.SQUARROSA (1043)/DOY1	1.90	MR	
293	**DVERD_2***	2.54	MR	13
294	DVERD_2/AE.SQUARROSA (1022)	1.32	R	
295	DVERD_2/AE.SQUARROSA (1026)	1.17	R	
296	DVERD_2/AE.SQUARROSA (1029)	1.22	R	
297	DVERD_2/AE.SQUARROSA (1031)	1.27	R	
298	DVERD_2/AE.SQUARROSA (214)	1.52	R	
299	DVERD_2/AE.SQUARROSA (221)	1.28	R	
300	DVERD_2/AE.SQUARROSA (247)	1.43	R	
301	DVERD_2/AE.SQUARROSA (247)	1.20	R	
302	DVERD_2/AE.SQUARROSA (333)	1.20	R	
303	DVERD_2/AE.SQUARROSA (507)	1.76	R	
304	DVERD_2/T.URARTU (545)	2.92	MS	
305	AE.SQUARROSA (1031)/DVERD_2	1.13	R	
306	AE.SQUARROSA (1029)/DVERD_2	1.77	MR	
307	**FALCIN_1***	1.50	R	5
308	FALCIN/AE.SQUARROSA (312)	1.35	R	
309	FALCIN/AE.SQUARROSA (389)	2.67	MS	
310	FALCIN_1/AE.SQUARROSA (1073)	2.06	MR	
311	FALCIN_1/AE.SQUARROSA (176)	1.78	MR	
312	FALCIN_1/AE.SQUARROSA (197)	1.63	MR	
313	**FGO/USA2111***	2.47	MR	1
314	FGO/USA2111//AE.SQUARROSA (658)	1.08	R	
315	**GAN***	1.06	R	39
316	GAN/AE.SQUARROSA (1080)	1.23	R	
317	GAN/AE.SQUARROSA (163)	1.20	R	
318	GAN/AE.SQUARROSA (180)	1.27	R	
319	GAN/AE.SQUARROSA (182)	1.62	MR	
320	GAN/AE.SQUARROSA (201)	1.92	MR	
321	GAN/AE.SQUARROSA (206)	2.13	MR	

322	GAN/AE.SQUARROSA (231)	2.10	MR
323	GAN/AE.SQUARROSA (233)	1.14	R
324	GAN/AE.SQUARROSA (257)	1.02	R
325	GAN/AE.SQUARROSA (264)	1.21	R
326	GAN/AE.SQUARROSA (267)	2.15	MR
327	GAN/AE.SQUARROSA (268)	2.26	MR
328	GAN/AE.SQUARROSA (285)	1.29	R
329	GAN/AE.SQUARROSA (296)	1.24	R
330	GAN/AE.SQUARROSA (300)	1.58	MR
331	GAN/AE.SQUARROSA (335)	1.00	R
332	GAN/AE.SQUARROSA (408)	1.02	R
333	GAN/AE.SQUARROSA (413)	1.11	R
334	GAN/AE.SQUARROSA (446)	2.27	MR
335	GAN/AE.SQUARROSA (459)	1.54	R
336	GAN/AE.SQUARROSA (479)	1.32	R
337	GAN/AE.SQUARROSA (522)	1.86	MR
338	GAN/AE.SQUARROSA (536)	1.87	MR
339	GAN/AE.SQUARROSA (620)	1.28	R
340	GAN/AE.SQUARROSA (621)	1.48	R
341	GAN/AE.SQUARROSA (623)	1.04	R
342	GAN/AE.SQUARROSA (624)	1.06	R
343	GAN/AE.SQUARROSA (633)	1.00	R
344	GAN/AE.SQUARROSA (638)	1.02	R
345	GAN/AE.SQUARROSA (643)	1.38	R
346	GAN/AE.SQUARROSA (658)	1.08	R
347	GAN/AE.SQUARROSA (668)	1.23	R
348	GAN/AE.SQUARROSA (680)	1.35	R
349	GAN/AE.SQUARROSA (721)	2.29	MR
350	GAN/AE.SQUARROSA (735)	1.36	R
351	GAN/AE.SQUARROSA (741)	1.00	R

352	GAN/AE.SQUARROSA (768)	1.13	R	
353	GAN/AE.SQUARROSA (779)	1.78	MR	
354	GAN/AE.SQUARROSA (890)	1.15	R	
355	**GARZA/BOY***	2.34	MR	7
356	GARZA/BOY//AE.SQUARROSA (271)	1.33	R	
357	GARZA/BOY//AE.SQUARROSA (286)	2.39	MR	
358	GARZA/BOY//AE.SQUARROSA (307)	1.21	R	
359	GARZA/BOY//AE.SQUARROSA (311)	2.26	MR	
360	GARZA/BOY//AE.SQUARROSA (350)	1.26	R	
361	GARZA/BOY//AE.SQUARROSA (439)	2.27	MR	
362	GARZA/BOY//AE.SQUARROSA (764)	1.98	MR	
363	**GREEN_3***	1.19	R	1
364	GREEN/AE.SQUARROSA (458)	1.00	R	
365	**KAPUDE_1***	2.13	MR	1
366	KAPUDE/AE.SQUARROSA (175)	1.88	MR	
367	**LARU***	2.31	MR	4
368	LARU/AE.SQUARROSA (309)	1.00	R	
369	LARU/AE.SQUARROSA (309)	1.00	R	
370	LARU/AE.SQUARROSA (333)	1.18	R	
371	LARU/AE.SQUARROSA (TA2459)	1.41	R	
372	**LCK59.61***	3.18	MS	2
373	LCK59.61/AE.SQUARROSA (308)	1.23	R	
374	LCK59.61/AE.SQUARROSA (783)	3.47	MS	
375	**LOCAL RED***	2.90	MS	7
376	LOCAL RED/AE.SQUARROSA (189)	2.20	MR	
377	LOCAL RED/AE.SQUARROSA (219)	2.64	MS	
378	LOCAL RED/AE.SQUARROSA (220)	1.80	MR	
379	LOCAL RED/AE.SQUARROSA (221)	2.40	MR	
380	LOCAL RED/AE.SQUARROSA (222)	3.04	MS	
381	LOCAL RED/AE.SQUARROSA (223)	2.19	MR	

382	LOCAL RED/AE.SQUARROSA (449)	1.42	R	
383	**RABI//GS/CRA***	1.63	MR	4
384	RABI//GS/CRA/3/AE.SQUARROSA (190)	2.01	MR	
385	RABI//GS/CRA/3/AE.SQUARROSA (457)	1.54	R	
386	RABI//GS/CRA/3/AE.SQUARROSA (891)	1.08	R	
387	RABI//GS/CRA/3/AE.SQUARROSA (904)	1.57	MR	
388	**RASCON_37***	2.18	MR	2
389	RASCON/AE.SQUARROSA (312)	1.08	R	
390	RASCON/AE.SQUARROSA (367)	1.44	R	
391	**ROK/KML***	2.72	MS	4
392	ROK/KML//AE.SQUARROSA (214)	1.65	MR	
393	ROK/KML//AE.SQUARROSA (295)	2.03	MR	
394	ROK/KML//AE.SQUARROSA (333)	2.27	MR	
395	ROK/KML//AE.SQUARROSA (507)	2.70	MS	
396	**SCAUP***	3.85	S	3
397	SCA/AE.SQUARROSA (248)	2.90	MS	
398	SCA/AE.SQUARROSA (409)	1.58	MR	
399	SCA/AE.SQUARROSA (493)	1.98	MR	
400	**SCOOP_1***	1.06	R	3
401	SCOOP_1/AE.SQUARROSA (358)	1.03	R	
402	SCOOP_1/AE.SQUARROSA (407)	1.00	R	
403	SCOOP_1/AE.SQUARROSA (659)	1.00	R	
404	**SCOT/MEXI_1***	2.35	MR	1
405	SCOT/MEXI_1//AE.SQUARROSA (186)	1.84	MR	
406	**SHAG_22***	1.50	R	6
407	SHAG_22/AE.SQUARROSA (1101)	1.20	R	
408	SHAG_22/AE.SQUARROSA (227)	1.59	MR	
409	SHAG_22/AE.SQUARROSA (319)	1.67	MR	
410	SHAG_22/AE.SQUARROSA (530)	1.33	R	
411	SHAG_22/AE.SQUARROSA (537)	1.55	MR	

412	SHAG_22/AE.SQUARROSA (539)	1.47	R	
413	**SNIPE/YAV79//DACK/TEAL***	1.53	R	7
414	SNIPE/YAV79//DACK/TEAL/3/AE.SQUARROSA (411)	1.20	R	
415	SNIPE/YAV79//DACK/TEAL/3/AE.SQUARROSA (528)	1.02	R	
416	SNIPE/YAV79//DACK/TEAL/3/AE.SQUARROSA (628)	1.00	R	
417	SNIPE/YAV79//DACK/TEAL/3/AE.SQUARROSA (629)	1.25	R	
418	SNIPE/YAV79//DACK/TEAL/3/AE.SQUARROSA (633)	1.03	R	
419	SNIPE/YAV79//DACK/TEAL/3/AE.SQUARROSA (700)	1.08	R	
420	SNIPE/YAV79//DACK/TEAL/3/AE.SQUARROSA (904)	1.13	R	
421	**SORA***	3.38	MS	14
422	SORA/AE.SQUARROSA (191)	2.68	MS	
423	SORA/AE.SQUARROSA (192)	2.10	MR	
424	SORA/AE.SQUARROSA (192)	1.68	MR	
425	SORA/AE.SQUARROSA (207)	1.72	MR	
426	SORA/AE.SQUARROSA (208)	2.49	MR	
427	SORA/AE.SQUARROSA (211)	1.28	R	
428	SORA/AE.SQUARROSA (215)	1.25	R	
429	SORA/AE.SQUARROSA (323)	1.04	R	
430	SORA/AE.SQUARROSA (442)	1.13	R	
431	SORA/AE.SQUARROSA (469)	1.30	R	
432	SORA/AE.SQUARROSA (617)	1.15	R	
433	SORA/AE.SQUARROSA (625)	1.18	R	
434	SORA/AE.SQUARROSA (684)	1.39	R	
435	SORA/AE.SQUARROSA (939)	2.42	MR	
436	**STY,DR/CELTA//PALS/3/SRN_5***	2.68	MS	2
437	STY,DR/CELTA//PALS/3/SRN_5/4/AE.SQUARROSA (277)	1.72	MR	
438	STY,DR/CELTA//PALS/3/SRN_5/4/AE.SQUARROSA (502)	1.27	R	
439	**TK SN1081***	2.88	MS	3
440	TK SN1081/AE.SQUARROSA (222)	1.19	R	
441	TK SN1081/AE.SQUARROSA (222)	1.06	R	

442	TK SN1081/AE.SQUARROSA (690)	1.20	R	
443	**YAR***	3.66	S	4
444	YAR/AE.SQUARROSA (493)	1.77	MR	
445	YAR/AE.SQUARROSA (518)	1.33	R	
446	YAR/AE.SQUARROSA (783)	1.48	R	
447	YAR/AE.SQUARROSA (809)	1.04	R	
448	**YAV_2/TEZ***	2.94	MS	12
449	YAV_2/TEZ//AE.SQUARROSA (1093)	1.54	R	
450	YAV_2/TEZ//AE.SQUARROSA (249)	2.55	MR	
451	YAV_2/TEZ//AE.SQUARROSA (249)	2.22	MR	
452	YAV_2/TEZ//AE.SQUARROSA (249)	1.97	MR	
453	YAV_2/TEZ//AE.SQUARROSA (249)	1.82	MR	
454	YAV_2/TEZ//AE.SQUARROSA (249)	1.78	MR	
455	YAV_2/TEZ//AE.SQUARROSA (249)	1.10	R	
456	YAV_2/TEZ//AE.SQUARROSA (435)	1.11	R	
457	YAV_2/TEZ//AE.SQUARROSA (437)	1.13	R	
458	YAV_2/TEZ//AE.SQUARROSA (721)	2.02	MR	
459	YAV_2/TEZ//AE.SQUARROSA (746)	1.03	R	
460	YAV_2/TEZ//AE.SQUARROSA (882)	1.00	R	
461	**YARMUK***	2.79	MS	4
462	YUK/AE.SQUARROSA (217)	1.61	MR	
463	YUK/AE.SQUARROSA (434)	1.10	R	
464	YUK/AE.SQUARROSA (784)	2.08	MR	
465	YUK/AE.SQUARROSA (864)	1.97	MR	
	**Lines without durum wheat parents in this study**			
466	KUCUK/AE.SQUARROSA (1080)	1.36	R	
467	KUCUK/AE.SQUARROSA (458)	1.03	R	
468	KUCUK/AE.SQUARROSA (640)	1.28	R	
469	DUKEM_12/2*RASCON_21//AE.SQUARROSA (1090)	1.07	R	
470	DUKEM_12/2*RASCON_21//AE.SQUARROSA (1100)	2.33	MR	

471	SRN/AE.SQUARROSA (358)	1.02	R
472	CADO/BOOMER_33//AE.SQUARROSA (504)	2.18	MR
473	CADO/BOOMER_33//AE.SQUARROSA (651)	1.00	R
474	CADO/BOOMER_33//AE.SQUARROSA (949)	1.15	R
475	YAV79//DACK/RABI/3/SNIPE/4/AE.SQUARROSA (381)	1.48	R
476	YAV79//DACK/RABI/3/SNIPE/4/AE.SQUARROSA (397)	1.18	R
477	YAV79//DACK/RABI/3/SNIPE/4/AE.SQUARROSA (443)	1.19	R
478	YAV79//DACK/RABI/3/SNIPE/4/AE.SQUARROSA (460)	1.13	R
479	YAV79//DACK/RABI/3/SNIPE/4/AE.SQUARROSA (460)	1.05	R
480	YAV79//DACK/RABI/3/SNIPE/4/AE.SQUARROSA (477)	1.63	MR
481	YAV79//DACK/RABI/3/SNIPE/4/AE.SQUARROSA (477)	1.48	R
482	YAV79//DACK/RABI/3/SNIPE/4/AE.SQUARROSA (490)	1.20	R
483	BACANORA T 88	1.83	MR
	Check resistant (Erik)	1.00	R
	Check susceptible (Glenlea)	4.80	S
	Check moderately resistant (6B-662)	2.50	MR
	Check moderately susceptible (6B-365)	3.40	MS

* Durum wheat parents.

**Averaged tan spot reaction of each genotype of SHW (twelve replications) and durum wheat parents (eight replications)

Supplementary Table S2. Candidate genes for significant marker-trait associations identified from *Triticum aestivum* (IWGSC), *Triticum turgidum* (Svevo.v1), *Aegilops tauschii* (Aet_v4.0), and *Triticum dicoccoides* (WEWSeq_v.1.0). Data was obtained from Emsembl https://plants.ensembl.org/

**Chromosome**	**Marker**	**Gene**	**Description**
1D	3026113	AET1Gv20669700	-
2D	1046601	TraesCS2D02G432700	-
5A	1200982	TraesCS5A02G23860	-
		TRITD5Av1G148960	Galactoside 2-alpha-L-fucosyltransferase
5A	3064590	TraesCS5A02G254500	-
		TRITD5Av1G155700	F-box family protein
6A	1862737	TraesCS6A02G378800,	-
		TRITD6Av1G217060	Cytochrome P450
6A	100027398	TraesCS6A02G381900	-
		TRITD6Av1G217800	F-box protein PP2
6A	2266481	TraesCS6A02G384200	-
7D	16793126	TraesCS7D02G203900	-
		AET7Gv20511100	-
		AET7Gv20511200	-
7D	993425	TraesCS7D02G524200	-
		AET7Gv21298500	-

Supplementary Figure S1. Principal component analysis of the synthetic wheat panel used in this study.

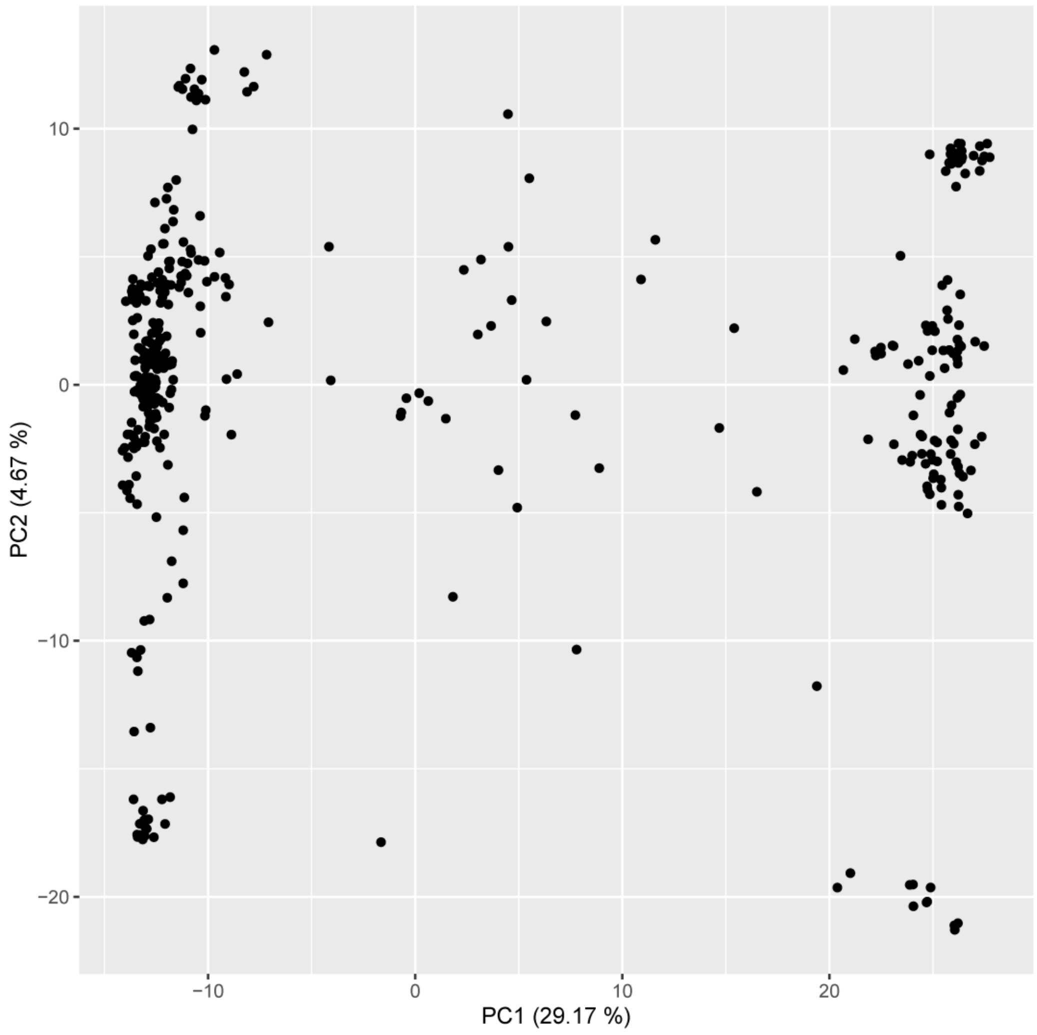

Supplement: Supplementary file 1 [file plants-11-00433-s001.zip › plants-1569676-supplementary.pdf]
